# Implications of recurrent disturbance for genetic diversity

**DOI:** 10.1002/ece3.1948

**Published:** 2016-01-25

**Authors:** Ian D. Davies, Geoffrey J. Cary, Erin L. Landguth, David B. Lindenmayer, Sam C. Banks

**Affiliations:** ^1^ The Fenner School of Environment and SocietyThe Australian National UniversityCanberraACTAustralia; ^2^ Division of Biological SciencesUniversity of MontanaMissoulaMontana

**Keywords:** Allele surfing, disturbance regimes, range expansion, recolonization, simulation

## Abstract

Exploring interactions between ecological disturbance, species’ abundances and community composition provides critical insights for ecological dynamics. While disturbance is also potentially an important driver of landscape genetic patterns, the mechanisms by which these patterns may arise by selective and neutral processes are not well‐understood. We used simulation to evaluate the relative importance of disturbance regime components, and their interaction with demographic and dispersal processes, on the distribution of genetic diversity across landscapes. We investigated genetic impacts of variation in key components of disturbance regimes and spatial patterns that are likely to respond to climate change and land management, including disturbance size, frequency, and severity. The influence of disturbance was mediated by dispersal distance and, to a limited extent, by birth rate. Nevertheless, all three disturbance regime components strongly influenced spatial and temporal patterns of genetic diversity within subpopulations, and were associated with changes in genetic structure. Furthermore, disturbance‐induced changes in temporal population dynamics and the spatial distribution of populations across the landscape resulted in disrupted isolation by distance patterns among populations. Our results show that forecast changes in disturbance regimes have the potential to cause major changes to the distribution of genetic diversity within and among populations. We highlight likely scenarios under which future changes to disturbance size, severity, or frequency will have the strongest impacts on population genetic patterns. In addition, our results have implications for the inference of biological processes from genetic data, because the effects of dispersal on genetic patterns were strongly mediated by disturbance regimes.

## Introduction

The importance of ecological disturbance as a driver of biodiversity patterns is being increasingly recognized as disturbance regimes change globally (Turner 2010). Global change is influencing the frequency, intensity, seasonality, and spatial patterns of ecological disturbances such as wildland fires, floods, and hurricanes (Franklin and Forman 1987; Webster et al. 2005; Boer et al. 2009; Cary et al. 2012). By definition, disturbance events cause temporal fluctuations in the distribution and abundance of species by altering niche opportunities (Shea et al. 2004), with outcomes strongly depending on the event type, species life‐history strategies (Noble and Slatyer 1980; Romiguier et al. 2014) and their phenotypic plasticity (Anderson et al. 2012). Repeated disturbances through time, otherwise known as the disturbance regime (Gill 1975; Krebs et al. 2010), critically influence species richness and composition within ecological communities (Connell and Slatyer 1977; Sousa 1984; Foster et al. 1998; Shea et al. 2004; Miller et al. 2011; Gill et al. 2013). However, the manner in which disturbance regimes affect genetic diversity is less well‐understood (Banks et al. 2013). The possible influence of disturbance regimes on the spatial and temporal patterns of genetic diversity has potential ramifications for the viability of populations, the adaptability of species (Lowe and Allendorf 2010), and for inferences made about the underlying mechanisms driving landscape genetic structure.

Disturbance regimes are classically considered as the history of disturbance at a point (Gill 1975; Krebs et al. 2010). This history is a record of components of the disturbance itself without reference to antecedent or postdisturbance conditions (Feller 1996; Keeley 2009; Spies et al. 2012). While these components may differ between disturbance types, for fire, measures of intensity (Byram 1959), interdisturbance interval, and the season of occurrence have relevance for biological outcomes (Gill 1975). These measures can also be applied to other disturbance types such as grazing (Koerner and Collins 2014), floods (Thodsen et al. 2014), pests (Raffa et al. 2008), and storms (Lirman 2003). While debate exists as to whether disturbance size (area) should be included as a component of disturbance regimes (Krebs et al. 2010) it could be assumed that larger disturbances will have demographic and genetic implications by more readily isolating populations or by mediating the impacts of colonization processes on spatial genetic patterns (Hallatschek et al. 2007).

Disturbance can influence genetic patterns through both selection‐driven and neutral processes. For instance, fire regimes influence the evolution of flammability‐enhancing traits in the shrub *Ulex parvoflorus* (Moreira et al. 2014). Disturbance regimes can also act as selective “filters” when related species share traits that influence their susceptibility to, or requirement of, disturbance (Helmus et al. 2010). Most case studies of the impacts of disturbance on neutral genetic diversity, although conducted in the context of an historical disturbance regime, have specifically focussed on quantifying impacts of single disturbance events only. A synthesis of these studies reveals that genetic effects are influenced by disturbance severity (e.g., the number or proportion of survivors in disturbed populations) and dispersal (Banks et al. 2011). High severity disturbance events can have long‐lasting effects on neutral genetic diversity in isolated populations (Beheregaray et al. 2003), but little impact when rates of in situ survival or postdisturbance immigration are high (Spear and Storfer 2010; Suárez et al. 2012; Banks et al. 2015).

While the genetic impacts of disturbance regimes can be considered as cumulative impacts of past events, it is important to recognize that the genetic outcomes of any one disturbance are contingent on disturbance history at that location. Thus, a diversity of potential states exist before any particular disturbance event (Hughes and Connell 1999); states that represent different distribution and abundance of individuals, and diversity and spatial structure of genes. This diversity, within and around the disturbed area, provides the source of migrants and hence the demographic and genetic context for recovery. Just as Connell (1980) notes the importance of the “ghost of competition past” in understanding the diversity and coevolution of competing species, we argue that patterns of genetic diversity in the landscape should be examined in the context of disturbance history.

A history of theoretical research on the genetic outcomes of extinction‐recolonization dynamics in metapopulations (one of many circumstances that can arise from disturbance regimes) provides a starting point for developing expectations about the impacts of disturbance regimes (Slatkin 1977; Wade and McCauley 1988; Whitlock and McCauley 1990; Pannell and Charlesworth 2000). Much of this research stems from the seminal work of Slatkin (1977) who considered the diversity of migrant sources and population turnover times (spatially uncorrelated single‐colony extinction and recolonization events) as critical considerations in determining total metapopulation diversity (*H*_T_), diversity within demes (*H*_S_) and differentiation among demes (*F*_ST_). Slatkin (1977) identified the differing effects that the so called *propagule* and *migrant‐pool* models have on genetic differentiation using a stepping‐stone model, noting these as two extremes of a continuum that exists in nature. A separate avenue of more recent research has shown that selectively neutral processes occurring during population expansion or colonization can lead to spatial genetic structure that can persist over long time periods (Hallatschek et al. 2007; Excoffier and Ray 2008; Waters et al. 2013). This leads to the expectation that genetic patterns resulting from historical contingency will shape genetic outcomes of disturbance events occurring within a long‐term regime.

To understand the potentially complex relationships that may exist between landscape patterns of genetic diversity and disturbance, we focus on modelling space, time, demography, disturbance, and genetics in a simple and neutral form (Gardner et al. 1987). We developed a simulation model with a key focus on ecologically relevant disturbance regime components that are expected to vary in response to climate change and environmental management (Seidl et al. 2011b; Bradstock et al. 2012). Using this model, we addressed a series of questions about the impacts and relative importance of these disturbance regime components on genetic diversity:

1. *How does the size and frequency of disturbance affect spatial and temporal patterns of genetic diversity across a landscape?* The frequency and size of wildland fire disturbance, for example, is expected to increase in many regions (Flannigan et al. 2009) and we expect disturbance size (i.e., spatially auto‐correlated patterns of disturbance events) to be of critical importance.
2. *How does variation in the intensity of disturbance mediate the effects of disturbance regimes on genetic diversity?* We consider disturbance as an agent of mortality, that is otherwise ecologically and genetically neutral, and represent effects of intensity (severity) by varying the degree of in situ survival. Extinction of entire populations within a disturbance footprint represents one extreme scenario of disturbance intensity (Slatkin 1977; Wade and McCauley 1988; Whitlock and McCauley 1990; Pannell and Charlesworth 2000). However, species have diverse mechanisms to persist in situ following disturbance events (Gill 1975; Whelan et al. 2002), and we expect that the impact of disturbance events on mortality will play an important role by influencing how predisturbance spatial patterns of genetic diversity are retained in postdisturbance landscapes, and by mediating the influence of recolonization on spatial genetic patterns.
3. *How do underlying patterns of dispersal and demography (birth rate) influence the interaction between disturbance regimes and genetic patterns?* We expect that these parameters will not only influence the patterns of recovery from specific disturbance events, but influence the pre‐existing spatial genetic context in which recovery occurs.

The novelty of this study was to use simulation to understand the relative importance of variation in key components of disturbance regimes and demographic processes, in driving landscape patterns of genetic diversity. Insights of this nature address a key gap in understanding disturbance‐driven ecological genetics (Banks et al. 2013), and help to formulate hypotheses concerning possible indirect effects on landscape patterns of genetic diversity through altered disturbance regimes.

## Methods

### Conceptual approach

**Figure 1:**
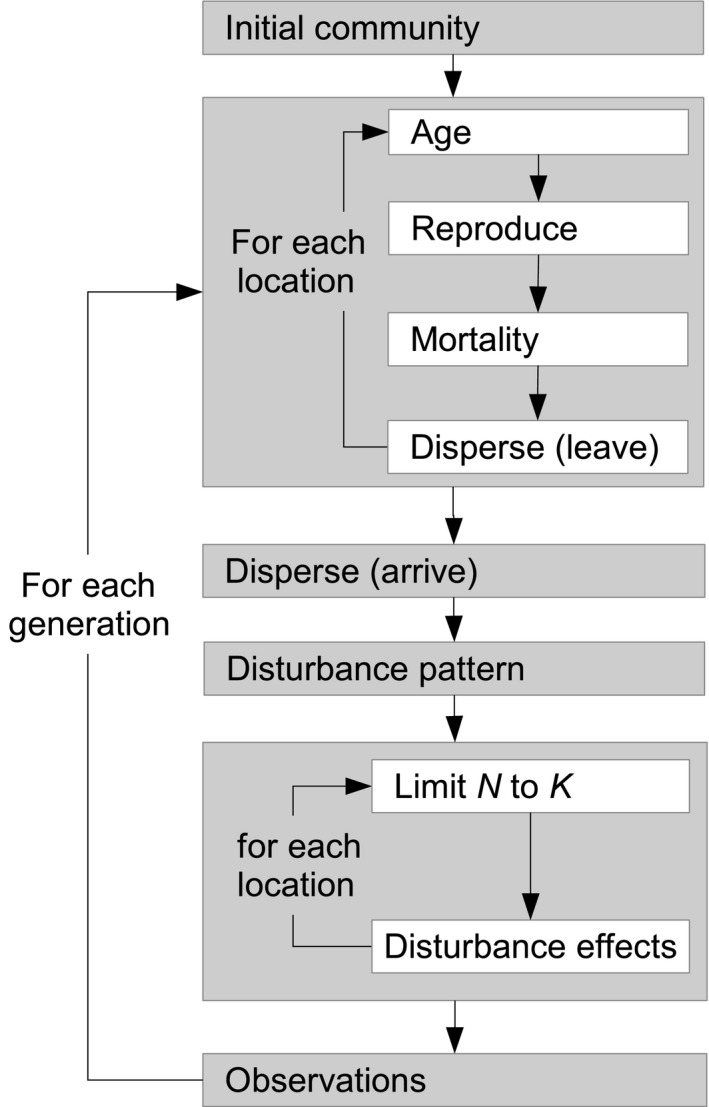
Flowchart for the genetics, demography and disturbance simulation model. Model details are fully described in the text and Appendix S2. Note that in the case of nonoverlapping generations, as used in this paper, the initial population comprises new born individuals only. These become adults during the “Age” process.

We developed a single‐species spatially explicit, individual‐based model (Fig. 1) to simulate five key processes that can potentially influence genetic diversity: (1) dispersal distance; (2) birth rate; (3) disturbance frequency; (4) disturbance size; and (5) severtiy of disturbance effects (rates of in situ survival) (Table 1). We used a generalized linear modeling design (ANOVA) (R Core Team, 2014) to examine the relative importance of these experimental factors in explaining variation in between‐ and within‐population genetic diversity. We limited our analysis to the relative proportion of variance explained by experimental factors and their interactions, rather than reporting statistical significance (Cary et al. 2006; White et al. 2014). We also quantified the spatial pattern of genetic differentiation among subpopulations (isolation‐by‐distance, IBD) resulting from different disturbance scenarios.

**Table 1:** Five experimental treatments used to evaluate implications of recurrent disturbance for genetic diversity. Twelve replicate simulations were performed for each unique permutation of the factors producing 2916 simulations. Each simulation began with a 4000 generation “burn‐in” period and a further 1000 generations were performed to produce model outputs for analysis

Factor	Levels	Description
Mean dispersal distance (MD)	Three	0.25, 0.5, or 1.0 cell units
Disturbance size (DS)	Three	Squares of 6, 12, or 24 cell units in width
Disturbance interval (DI)	Three	Mean frequency 30, 60, or 120 years
Disturbance severity (SV)	Three	100%, 95%, or 85% of individuals removed from cells within the disturbance perimeter
Birth rate (BR)	Three	3, 6, or 12 individuals produced by each female (unless males are absent)

### Landscape

We used a gridded 102 × 102 cell homogeneous landscape with each cell assigned the same carrying capacity (*K*), after noting that preliminary simulations on a 204 × 204 cell landscape yielded similar final results (Appendix S1). To ensure a sufficiently large sample size for analysis, the 102 × 102 cell landscape was divided into 1156 sample populations, each comprising a square of nine grid cells. A sample population, therefore, has a carrying capacity of 9 × 20 (180 individuals). Preliminary simulations with larger values of *K* did not substantially change the final results but did require simulating a greater number of generations (Appendix S1). All spatial processes (dispersal and disturbance) were isotropic, as heterogeneous cost‐surfaces for mate selection and dispersal were not invoked. This provides the opportunity to employ a topologically infinite landscape; a torus topology (Haefner et al. 1991), which provides three key benefits: (1) measures of genetic difference between populations by distance do not suffer a sampling bias; (2) edge effects can be avoided; and (3) populations simulated on an infinite landscape are fourfold larger than a finite landscape of the same size, reducing the occurrence of populations that are too small to provide rigorous inferences. Importantly, our results using a finite landscape did not show any important differences in the model outputs examined here (Appendix S1). We chose to present the results of the infinite landscape model to focus on parameter sensitivity and interaction without the added complexity that model behavior may exhibit at absorbent and reflective boundaries in finite landscapes.

### Demography

A variable population (*N*) of size 0 to *K* exists at any time in each cell, with *K* set to 20 (see Appendix S1 for discussion of model sensitivity to *K*). We initialized the simulations with *N *= *K* new‐born individuals per cell with a 50:50 sex ratio. Mating and demographic structure parameters were set to represent a population that was dioecious. Both females and males mate with replacement, and generations were nonoverlapping. We assumed the population growth rate was female‐limited (eq. (1)):

(1) $$N_{\left(t+1\right)}=\left[N_{\left(f,t\right)}2\lambda +D_{i}-D_{e}-D_{m}\right]$$

where: *N*_(*t* + 1)_ is the population at time *t* + 1; *N*_(*f*,* t*)_ is the number of females at time *t; λ* is the density‐independent population growth rate; *D*_i_ and *D*_e_ are the number of immigrants and emigrants, respectively; and *D*_m_ is the additional mortality due to disturbance. If the population exceeded *K*, individuals were removed by random selection from the simulation after each generation was processed but before disturbance (Fig. 1). Note that we assume disturbances occur late in the annual cycle and we do not examine the effects of the seasonality of disturbances. Furthermore, for some species, limiting population to carrying capacity after mortality rather than after dispersal may be more appropriate (Fig. 1). However, analysis of this alternative life‐cycle did not change the findings of this study (Appendix S1).

A density‐independent stepping‐stone dispersal model was used. The dispersal distance of each individual was calculated using a uniform random distance (*ε*), a value between 0–1, around a specified mean (*m*) (eq. (2)). Equation (2) was applied to both coordinates in two dimensions and *d* was rounded to integer values with a probability based on the fractional part of the coordinate.

(2) d=m(2ε−1)

For our simulation experiments, we considered three levels of mean dispersal distance (0.25, 0.5, or 1.0 cell units). This parameterization constrained dispersal to a stepping‐stone model in two dimensions where mean distance can be directly converted to dispersal rates as has been common in earlier studies (Slatkin 1977). For example, a mean dispersal distance of one cell corresponds to 25% of the dispersing individuals remaining within the source cell, 12.5% arriving at each of the cardinal neighbors and 6.25% dispersing to each of the intercardinal neighbors: equivalent to a migration rate of 75%. The equivalent dispersal rates for mean dispersal distances of 0.25 and 0.5 cells were 23.4% and 43.7% respectively. We compared this method with a negative exponential function (which may be more realistic in an IBD landscape) in Appendix S1. A negative exponential dispersal method enables some individuals to disperse beyond the immediate neighbors (20% if mean is one cell) as the maximum distance is unbounded. For the purpose of comparison, we describe dispersal in terms of *mean dispersal distance* rather than *dispersal rates* but provide the equivalent values above.

### Genetics

We used a neutral *k*‐allele model (*k *=* *10) (see Appendix S1 for discussion of the sensitivity of model results to *k*). Each individual had a diploid genotype with ten unlinked loci to approximate the number of loci and degree of polymorphism observed in many empirical microsatellite‐based studies. Mutation rate was constant at 10^−4^ per generation for each allele selection, which is near the mid‐range of mammalian microsatellite mutation rates (Jarne and Lagoda 1996). Initial allele assignment at each loci was chosen at random (Gibbs 2001; van Strien et al. 2015) from the ten alleles available for each locus, such that simulations began with an expected heterozygosity of 0.9.

### Disturbance

Disturbance patterns were described by variation in three factors: (1) disturbance size, (2) disturbance interval; and (3) minimum disturbance severity (mortality due to disturbance). Disturbances were arbitrarily square in shape with three levels of size (6, 12, or 24 cells wide). The place and time of each disturbance was uniformly random. Sufficient events of the specified size were generated to produce a mean interdisturbance interval (at each cell) of either 30, 60, or 120 generations for the three treatment levels. No constraint was imposed on the minimum interval between disturbances. The severity of disturbance effects can vary randomly between cells from 100% down to a prescribed minimum (100%, 95%, or 85% of individuals killed by disturbance). For any single simulation, disturbance size is held constant. We acknowledge that disturbances commonly occur with intensities or sizes that have a log distribution (Cumming 2001). Furthermore, their frequency often has a nonuniform distribution (Boer et al. 2008). However, we have avoided using typical distributions of disturbance size and frequencies to unambiguously examine the relative importance of these two measures. Nevertheless, we examined the consequences of relaxing this constraint in Appendix S1, but found no change from the results presented here.

### Simulation experiment

The simulations proceeded according to Fig. 1, and the experimental design encompassed all treatment permutations of variation in disturbance regime and demography outlined in Table 1. Overall, we considered five factors, each with three levels (Table 1). The level of each factor doubled the value of the previous level, which allowed us to efficiently span a wide domain and capture the shape of the response variables over treatment levels. We also performed 12 replicate simulations without disturbance to provide a baseline for IBD analysis.

Analysis of variance is necessarily sensitive to the range over which treatment levels are manipulated. In many cases, reference to real‐world circumstances will provide a guide to a realistic range. However, in the case of a neutral model, which does not specify any particular species, location or disturbance type, such choices risk being arbitrary. Ecological research has shown that the scale‐dependence of recovery processes (influenced by the rate of in situ survival and dispersal/recolonization capacity) mediates the sensitivity of population persistence to disturbance size and frequency (Romme et al. 1998). We grounded this experiment by bounding the treatment range at one extreme by choosing values that allow the population to become low, but not extinct, over the course of the 5000 generations. At the other extreme, we chose treatment levels beyond which effects were negligible. As a guide, it takes 60 generations for the population to fully recolonize an area impacted by our largest, high severity disturbance (24 × 24 cells) using the most restrictive dispersal and demographic scenario (a birth rate of 3 and a dispersal distance of 0.25). Total abundance over the landscape for this scenario is approximately 25% of *K*. Conversely, the same process took only 12 generations under our scenario with highest dispersal and birth rate (a birth rate of 12 and a dispersal distance of 1, resulting in a 68% occupancy rate). Viable populations might also occur if disturbance size is traded against frequency, and this may be important when considering the generality of our results. However, increasing birth rate beyond 12 will have no effect because: (1) the diversity of source populations does not change in a stepping‐stone model (as used here); and (2) birth rates beyond those used here, even when source populations are very small, will result in a population at carrying capacity at the first generation. Therefore, when considering scale and the application of this general study to particular cases, it is the relativity between carrying capacity and disturbance size that should be kept in mind. To confirm that the disturbance parameters are within a long‐term equilibrium we examined our parameter space using two metrics (*T* and *S*) used to define degrees of landscape equilibrium (Turner et al. 1993). *T* is the mean disturbance interval divided by the recovery time and *S* is the disturbance area divided by the landscape size. The range of *T* and *S* for the above two extreme cases define a rectangle which lies on the border of “equilibrium or steady state” and “stable, low variance” landscapes as defined by Turner et al. (1993). Choosing lower frequency and larger disturbance sizes would be a viable alternative, (type D in Turner et al. (1993)), however either proportionally longer simulations would be required (or more replicates) to limit variance in model outputs to an acceptable level.

While there are no relevant analytical disturbance models for comparison, the basic demographic model used here, when simulated under panmixia, results in patterns of genetic drift expected under Wright's formulation of $H_{T}={\left(1-\left(1/2N\right)\right)}^{t}\;H_{0}$. Thus, our underlying demographic‐genetic model “delivers” the expectations of population genetic theory, and the dispersal scenarios that we simulate are sufficiently simple and general that they can be representative of, or relevant to, many real scenarios.

### Statistical analysis of simulation results

Preliminary simulations of 10,000 generations (Appendix S1, Fig. 6), indicated that *H*_T_, $\bar{H{}_{S}}$, and *F*_ST_ were relatively stable after 4000 generations. Therefore, each simulation was preceded by a 4000 generation burn‐in with a further 1000 generations executed to provide data for analysis. We ran 12 replicates for each simulation resulting in 2916 runs. Replicates differ only in their random number seeds which affect: (1) initial allele assignment; (2) Mendelian genetics and mutation; (3) disturbance time and location; (4) distance and direction of individual dispersal events; and (5) random post‐disturbance survival. We used only 12 replicates per scenario because the large landscapes (up to approximately 2 × 10^5^ individuals per generation) resulted in little variation among replicates within treatments. Indeed, the coefficient of variation in *F*_ST_ between replicates (averaged over the last 1000 generations) was, at most, 18% for simulations with large, frequent and severe disturbances with low dispersal distance and low growth rates (i.e., scenarios with the greatest stochastic variability).

For a given population, heterozygosity was averaged over the number of loci *l* for frequency *p* of allele *i* of *k*.

(3) $$H=\frac{1}{l}\sum_{j=1}^{l}\left(1-\sum_{i=1}^{k}p_{i}^{2}\right)$$

The average heterozygosity of populations ($\bar{H{}_{S}}$) was the sum of *H*_i_, weighted by the population size (*N*_i_):

(4) $$\bar{H{}_{S}}=\frac{\sum_{i=1}^{s}H_{i}N_{i}}{\sum_{i=1}^{s}N_{i}}$$

The measure of genetic difference between populations, where *H*_T_ was determined using eq. (3) for the entire population, was:

(5) $$F_{\mathrm{ST}}=\frac{H_{T}-\bar{H{}_{S}}}{H_{T}}$$

In addition, 50 sample populations with *N *>* *0.1 *K* (18 individuals in nine cells) were randomly selected for pairwise *F*_ST_ analysis at the end of each simulation, to quantify spatial genetic structure arising from the experiments. Pairwise *F*_ST_ for populations *A* and *B* is given by (Nei 1978):

(6) $$F_{\mathrm{ST}}\left(AB\right)=\frac{H_{T}-\left(H_{A}N_{A}+H_{B}N_{B}\right)/\left(N_{A}+N_{B}\right)}{H_{T}}$$

We quantified the regression of *F*_ST_/(1–*F*_ST_) and log‐transformed geographic distance between populations, as suggested by Rousset (1997), and used distograms and Mantel correlograms to explore the effects of disturbance regimes on spatial patterning of the magnitude of *F*_ST_ and the *F*_ST_ by distance correlation, respectively, using the methods described in Diniz‐Filho et al. (2013).

## Results

### Effects of disturbance regimes on overall genetic diversity and differentiation

**Figure 2:**
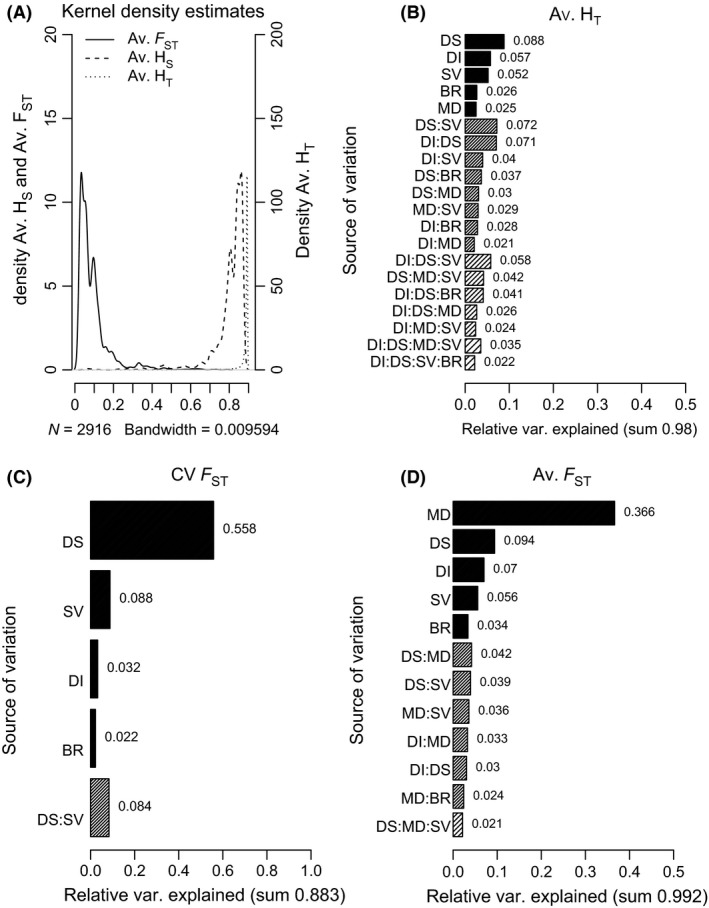
Comparison of (A) kernel density estimates of *H*_T_, *F*_ST_ and *H*_S_ from all scenarios together with analysis of the relative variation in three response variables explained by demographic and disturbance factors. Response variables for ANOVA analysis (R Core Team 2014) are relative variance explained for (B) Av. HT_T_, (C) temporal coefficient of variation of *F*_ST_ (over 1000 generations) and (D) Av *F*_ST_. For clarity, only factors and factor interaction explaining more than 2% of the variance are shown.

*H*_T_ was relatively unchanged between experimental treatments compared to $\left(\bar{H{}_{S}}\right)$ or *F*_ST_ (Fig. 2A). Variation in *H*_T_ was driven primarily by the disturbance variables that directly affect overall population size, especially disturbance size, including its interaction with birth rate and dispersal distance (Fig 2B). Variables that affect population size also contribute most to temporal variation in *F*_ST_ (CV*F*_ST_), predominantly disturbance size (56% of variance explained) (Fig. 2C). Because of the relative stability of H_T_, there was, by definition (eq. 5), a close inverse relationship between *F*_ST_ and $\left(\bar{H{}_{S}}\right)$ (Fig. 2A). For this reason, and because the major effects of disturbance were apparent in the spatial patterning of genetic diversity among subpopulations, we focused predominantly on the detail of *F*_ST_ in the results (Fig. 2D).

### Effects of disturbance regimes on spatial genetic structure (*F*_ST_)

**Figure 3:**
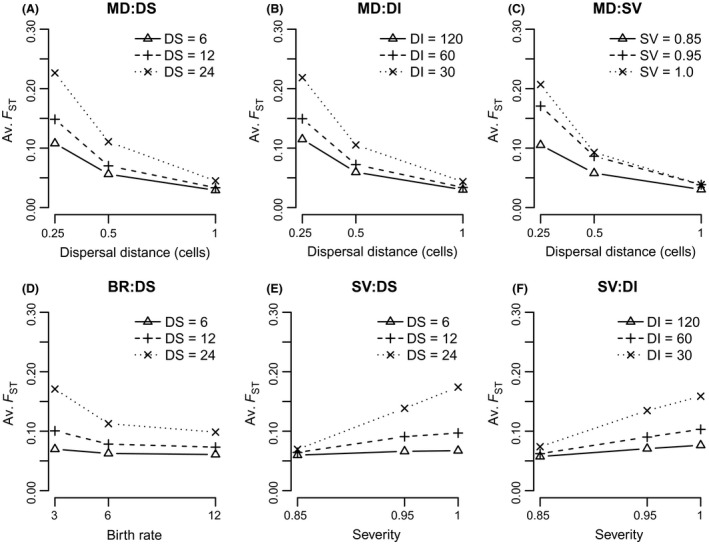
Two‐way interactions of disturbance treatments and mean dispersal distance (A, B, and C) of variation in *F*_ST_ (averaged over the last 1000 generations). Chart (D) shows the interaction of birth rate and disturbance size. Charts (E) and (F) show the interactions of disturbance severity with disturbance interval and size. These charts show values averaged over all parameters and replicates, except where otherwise indicated.

Variation in disturbance size (DS) explained around 9% of variation in simulated *F*_ST_, with only mean dispersal distance (MD) explaining greater variation (37%), while disturbance interval (DI) and severity explained 7% and 6% respectively (Fig. 2D). There were important interactions between the three disturbance variables, (size, frequency, and severity), and the mean dispersal distance (Fig. 2D). The effects of disturbance size and frequency were strong when dispersal distance was restricted to a mean of 0.25 cells, with both causing increases in genetic structure across the landscape, but the effects of DS and rates of in situ survial (SV) were negligible when MD was high (mean distance 1 cell) (Fig. 3A–C).

The least important parameter in our simulations was birth rate (BR) (Fig. 2D). While birth rate increases the rate at which the population can regenerate in disturbed areas, similar to the colonizing effect afforded by high dispersal distance, our results suggest that, within the treatment range, birth rate has a far lesser effect on *F*_ST_ in recovering populations, and therefore on genetic structure in the presence of disturbance.

Increasing in situ survival rates after disturbance (SV) markedly reduced any effect of disturbance on genetic structure, with disturbance having little effect on genetic structure under low severity (SV = 0.85) (Fig. 3E,F). Increasing disturbance interval (DI) decreased the effect of disturbance size in a linear fashion (not shown).

### Genetic isolation‐by‐distance

**Figure 4:**
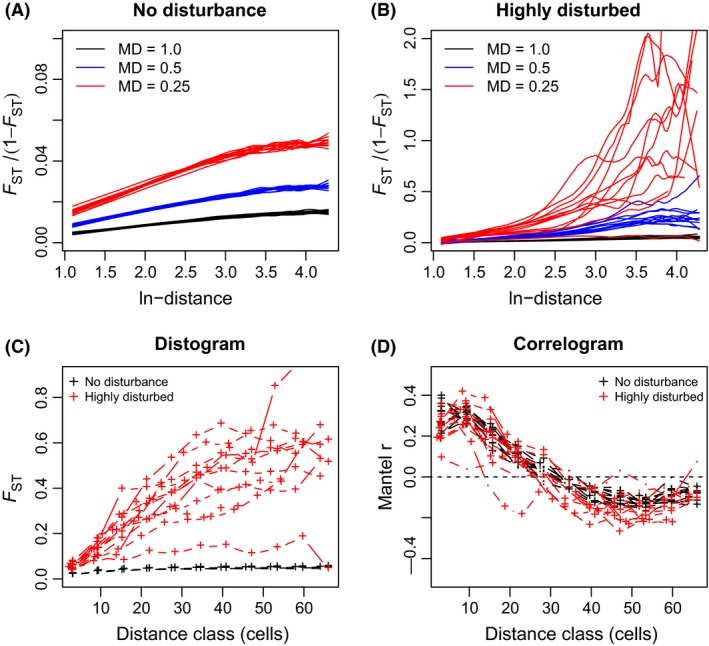
The relationship between transformed pairwise *F*_ST_ and log transformed distance for (A) undisturbed and (B) highly disturbed landscapes. For clarity, the scatter data for each replicate simulation is summarized as a Lowess regression (R Core Team). Chart (C) shows pairwise untransformed *F*_ST_ by distance class (Diniz‐Filho et al. 2013). In chart (D), significant values (*P* < 0.05) of correlation between genetic and spatial distance matrices are shown with a “+”, nonsignificant values are shown with open circles. The intersection of the dotted lines indicates the approximate patch size. These pairwise comparisons use 50, randomly selected sites at the end of each of the 12 replicate simulations. Consequently, there will be a greater variance between the 12 replicates shown here, than between the meta‐population *F*_ST_ averaged over 1000 generations used for analysis in Fig. 2. All examples use a birth rate of 3 and a dispersal distance of 0.25 unless otherwise indicated. For highly disturbed landscapes, the parameters are DI = 30 years; DS = 24 cells; SV = 1.

In the absence of disturbance in our model system, a quasi‐linear relationship existed between genetic and geographic distance when the data are transformed as *F*_ST_/(1–*F*_ST_) by *ln*‐distance (Fig. 4A). The slope of this relationship was sensitive to dispersal distance and also displayed a levelling off at a distance of log 35 (3.5) cells (Fig. 4A). However, this relationship was disrupted in simulations with high levels of disturbance and low dispersal distance, a situation where populations remain in fragmented patches for most of the simulation (Fig. 4B). The spread of *F*_ST_ across distance classes was greatly increased when disturbances were large, frequent and severe (Fig. 4C). However, we found Mantel correlograms more informative of IBD patterns that arose in the context of disturbance regimes than simply recording Mantel's *r* (Mantel 1967) (Fig 4D). In the absence of disturbance, the scale of positive autocorrelation was stable at a distance of approximately 30 cells (the patch size) across all treatments over the course of the simulations (Fig. 4D). However, for scenarios representing highly disturbed landscapes, while variability in Mantel's *r* by distance class was greatly increased, there was nevertheless a general trend of increasing correlation by distance class (both positive and negative) (Fig 4D).

### Temporal variability of *F*_ST_

**Figure 5:**
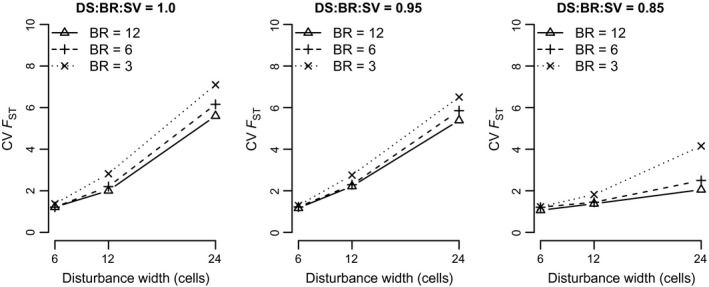
Temporal effects on *F*_ST_ of disturbance size, severity and birth rate. Charts show values averaged over all parameters and replicates, except where otherwise indicated.

The temporal coefficient of variation in *F*_ST_ (CV*F*_ST_), measured over the last 1000 generations of the simulation, was strongly affected by disturbance size, which explained around 56% of the variance in this response variable (Fig 2C). However, while higher birth rates decreased temporal variation of *F*_ST_, especially for the highest rate of in situ survival (Fig. 5), variance explained in CV*F*_ST_ by birth rate was still relatively minor compared to other parameters (Fig. 2C).

### General observations

**Figure 6:**
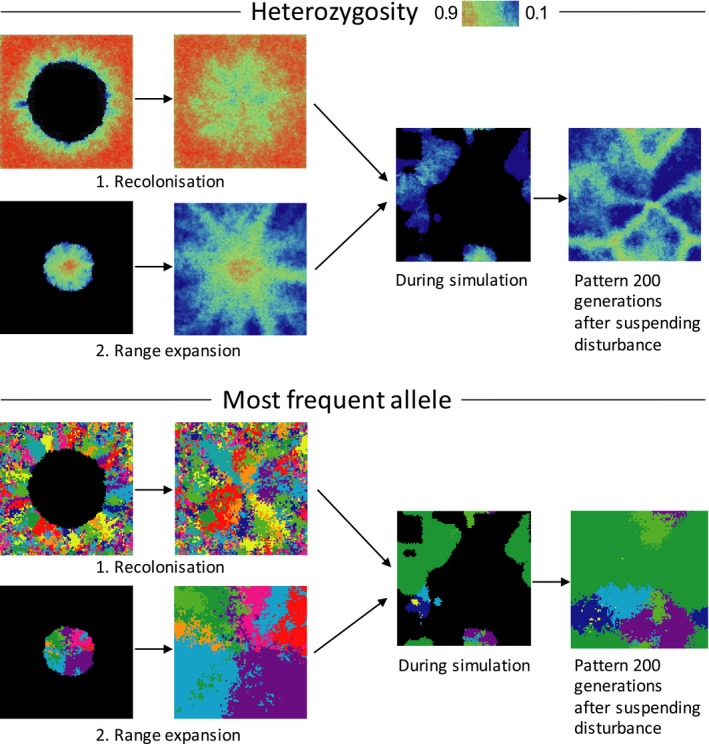
Spatial genetic patterns produced through a combination of range expansion and recolonization. On the left‐hand side of the figure are shown patterns of heterozygosity and the most frequent allele for (1) recolonization of an isolated empty area from a surrounding population and (2) range expansion from an isolated population into an empty landscape. In each case, the two processes in combination produce the patterns on the centre‐right during the simulation. The pattern is more apparent if disturbance is disabled for a period of time sufficient for the entire landscape to be fully occupied (far right).

Informally, for regimes incorporating large, frequent, and severe disturbances, our model produced a clumped patterns of high and low heterozygosity meditated by dispersal distance (Fig. 6). These patterns arose through a combination of two related pattern‐generating processes: *colonization* and *expansion*, whose impacts are mediated by disturbance severity. As used here, colonization was defined as spread from a surrounding population into vacant sites, while range expansion was the opposite; spreading out from isolated refugia into an empty landscape (Fig. 6), This led to patterns of genetic homogeneity (low heterozygosity) with zones of higher heterozygosity where colonization/expansion fronts meet. Once established, these patterns were relatively stable over time, and while the size of patches of genetic homogeneity were similar over replicates, each replicate was nevertheless unique in detail. The spatial covariance of variation in *H*_S_ showed an exponential increase with disturbance size. Using the most restrictive dispersal and demographic scenarios (a dispersal distance of 0.25 and a birth rate of 3), and the most severe and frequent disturbance (severity of 1 and mean interdisturbance frequency of 30 years), the spatial coefficient of variation in *H*_S_ increased from 2% where disturbance size was zero, to 54% when disturbance size was 24 cells in width. At the same time, mean *H*_S_ decreased by 84% from 0.82 to 0.13.

## Discussion

### Disturbance size and severity influence spatial genetic patterns

Disturbance regimes are critical attributes of ecosystems and can have large effects on community composition (Lake 2000; Berumen and Pratchett 2006; Lindenmayer et al. 2011). Here, we showed that disturbance regimes can have large impacts on spatial and temporal patterns of genetic structure. Of all disturbance variables, disturbance size and frequency had the greatest genetic effect in our model system. However, the amount of in situ survival, (“minimum severity” in our model), strongly mediates the overall genetic effects of disturbance (Fig. 4E,F). While the disturbance parameters tested here influenced the total level of genetic diversity to a small degree (Fig. 2B), their effect on genetic diversity within subpopulations was profound. Consequently, these disturbance regime components were associated with increases in the magnitude of genetic differentiation among populations (Fig. 2D) and changes in the spatial pattern of IBD (Fig. 4).

Several theoretical principles govern the genetic diversity of populations subject to extinction and recolonization events under idealized modes of colonization. Our study provides insights about when these theoretical principles are likely to apply under spatial and temporal variation in ecological disturbance patterns. For instance, population turnover rates are expected to increase *F*_ST_ under the *propagule pool* model (Pannell and Charlesworth 2000). This circumstance does not arise where disturbances are small or dispersal distance is large (relative to disturbance size), or in situ survivors are common (Gill 1975; Banks et al. 2011). However, recolonization is a process that operates in space, and our model suggests that the propagule pool model will predominate under regimes of severe disturbance when the disturbance footprint is large relative to dispersal distance. The scale of disturbance size and dispersal distance in our study ensures that this occurs even when the dispersal model is replaced with one that draws from a larger number of source populations (Appendix S1).

### Allele surfing and the role of population growth rate in spatial genetic patterns

We identified a likely role during disturbance regimes for the “allele surfing” process documented during range expansions (Hallatschek et al. 2007; Excoffier and Ray 2008). This was most apparent under scenarios of large disturbance size and high severity, where recolonization processes generated patches of genetic homogeneity, with high heterozygosity occurring in “mixing zones” between colonization fronts. Such patterns are not seen in undisturbed populations, but feature during biological invasions or extinction‐recolonization scenarios driven by disturbance regimes, long‐term climatic fluctuation and recolonization from refugia (Petit et al. 2003). In our model, this pattern is dynamic on a generational time scale, with refugia (populations surviving disturbance in situ) forming and re‐joining at a rate dependent on dispersal distance, disturbance size and the degree of in situ survival. The persistence of these patterns underlies the importance of the system state before each disturbance. Disturbances that span a region where two populations of low heterozygosity meet (a mixing zone) tend to recolonize with the same pattern of heterozygosity as existed before the disturbance, leaving relatively little indication of the footprint of the last disturbance. Having speculated that these patterns of heterozygosity, once established, appear relatively stable, future simulation studies may be able to quantify the stability of these patterns by some measure of their rate of change. Perhaps it is the case that, after establishing this “stable” state, the subsequent history of disturbance has, in fact, far less an effect on landscape genetic patterns than might be thought.

Our model system employed a neutral, density‐independent approach to rates of population growth. However, other research indicates that different outcomes could arise if this assumption were to be relaxed. The observed allele surfing patterns might not arise in cases where density‐dependent reproduction delays expansion from founding populations (Waters et al. 2013), or where gene flow is not tied to demographic expansion (pollen dispersal). Conversely, even greater rates of genetic drift during recolonization after severe disturbance will likely occur if a density‐dependent growth function were to be used that produced higher growth rates at low population densities (Waters et al. 2013).

The occurrence of allele surfing processes under regimes of large, severe disturbances may have implications for the inference of geographical variation selection from genome‐wide association studies. For instance, the occurrence of disturbance is commonly associated with spatial environmental variation (Swetnam and Betancourt 2010), but can generate strong spatial heterogeneity in genetic diversity through selectively‐neutral processes such as allele surfing (Excoffier and Ray 2008).

Our results provide insights into the conditions under which climate‐driven or landscape management‐driven shifts in disturbance patterns will have important consequences for genetic diversity, as influenced by selectively neutral demographic processes. Changes to disturbance size or frequency will have strong implications for genetic patterns when severity is high (i.e., there are few survivors) and dispersal capacity is restricted relative to the scale of disturbance. This finding has parallels with ecological research on the scale‐dependence of postdisturbance recovery processes, where the spatial dispersion of survivors and the scaling of colonization ability relative to disturbance area mediate the sensitivity of population distribution to disturbance size and return interval (Romme et al. 1998). Conversely, genetic patterns in species with long‐range dispersal capability (relative to disturbance size), may be insensitive to variation in disturbance size or severity. For many disturbance types, disturbance size and frequency may be positively correlated (such as wildland fire), and under such circumstances shifts in disturbance regimes from global change phenomena like climate change may have an enhanced role in determining spatial patterns of genetic diversity across landscapes. For example, King et al. (2013) have shown that climate change could increase area burned in mesic forested landscapes while at the same time reduce area burned in more arid grass dominated regions.

### Dispersal patterns mediate disturbance effects

Dispersal distance had a far stronger effect on $\bar{H{}_{S}}$ and *F*_ST_ than any of the disturbance parameters (Fig. 2D). Furthermore, dispersal distance strongly mediated the impacts of disturbance regimes on spatial genetic patterns because, for high dispersal rates, postdisturbance demographic recovery and genetic diversity were driven strongly by high immigration, irrespective of the presence of in situ survivors.

Under more realistic dispersal models (e.g., the negative exponential model in Appendix S1), higher dispersal scenarios imply that founders are drawn from larger, and therefore a more heterogeneous set of source populations, corresponding to the distinction between the *propagule pool* and *migrant pool* models (Pannell and Charlesworth 2000). However, when using a stepping‐stone model, the set of source populations can only vary between one and eight neighbors. Dispersal distance, therefore, directly determines the size of founding populations and it is the genetic drift that arises from this variation that drives the model (Hallatschek et al. 2007; Excoffier and Ray 2008). However, because we limit the population to *K* only after dispersal takes place (Fig. 1), the size of founding populations also depends on birth rate. While our finding that variation in genetic differentiation is more sensitive to dispersal distance than birth rate could be attributed to the range of treatments used for these two parameters, it should be noted that birth rate affects founding population size linearly. Dispersal distance, on the other hand, operates in two dimensions and the inverse square law ensures that its effect is nonlinear. For birth rate to be as sensitive as dispersal distance would require either an approximate threefold increase in birth rates or a halving of the maximum dispersal distance.

### Implications of spatial and temporal variability in genetic patterns

The spatial and temporal variability in *H*_S_ and *F*_ST_ resulting from disturbance regimes has some important implications for the measurement of neutral genetic patterns and the inference of biological processes. For instance, impacts of dispersal distance on *F*_ST_ were greater under scenarios of large and/or severe disturbance compared to undisturbed populations (Fig. 4A,B). For a given dispersal pattern, the relationships between pairwise *F*_ST_ and geographic distance were substantially different in the presence of strong disturbance compared to undisturbed populations. These changes were not represented by simple landscape‐wide Mantel correlation statistics (which test for the linear association between genetic and geographic distance), but by: (1) changes to overall *F*_ST_, Mantel *r* correlations within distance classes (which enables visualization of spatial variation in the association between genetic and geographic distance) (Fig. 4D), and (2) distograms, which allow exploration of changes in the magnitude of pairwise *F*_ST_ by distance class (Fig. 4C). The expected linear relationship between *ln*‐distance and *F*_ST_/(1–*F*_ST_) was apparent for undisturbed populations. However, even in IBD models of undisturbed populations, genetic drift will counter spatial patterns generated by gene flow at a certain distance threshold (van Strien et al. 2015). This is shown by our model (Fig. 4A), where at a distance of 35 cells, the transformed *F*_ST_ no longer responded to *ln*‐distance in the same manner. For scenarios in our model representing highly disturbed conditions, the linear IBD relationship is disrupted over much shorter geographic distances, indicating a stronger role of drift in driving spatial genetic patterns in disturbance‐prone ecosystems. Similar effects on IBD patterns (after single fire events) have been observed empirically in response to fire history in several species of Florida sand skink populations (Schrey et al. 2011). This presents problems for the inference of dispersal processes from spatial genetic data where disturbance history is unknown. However, the highly variable IBD results observed in Fig. 4B (MD = 0.25), occur only for scenarios which result in relatively low population numbers producing a spatially dynamic pattern of isolated populations (e.g., Fig. 6).

The temporal variability in $\bar{H{}_{S}}$ and *F*_ST_ driven by disturbance regimes (primarily disturbance size and severity [Fig. 2C]), highlights how interpretations of “snapshot” samples of genetic diversity need to consider this temporal variability when disturbance history is unknown. Under some conditions (e.g., extreme population isolation), the effects of single disturbance events can be observed in populations over millennial timescales (Beheregaray et al. 2003). Sampling designs conducted without knowledge of historical disturbance regimes may provide limited or misleading insights into contemporary processes (Dyer et al. 2010; Landguth et al. 2010). A valuable area of further research may involve the use of simulation tools to provide insights into the ability of empirical sampling designs to recover the known patterns of genetic diversity and dynamics from a simulation model. Simulation tools (possibly in an Approximate Bayesian Computation framework) may help with this. Indeed, such model‐based inference is increasingly common in molecular ecology, so considering disturbance scenarios in the ecological‐demographic‐genetic models used for these purposes is a logical approach to dealing with this problem.

### Toward increasing complexity

There is considerable scope for greater complexity in representation of key mechanisms in future studies, including representation of ecological, disturbance and genetic processes.

#### Ecological representation

Successional dynamics are a well‐documented consequence of disturbance (Bengtsson et al. 2000; Lake et al. 2007; Leavesley et al. 2010; Banks et al. 2011), with species commonly showing preference for early or late postdisturbance stages, or having habitat suitability mediated by disturbance return intervals. Therefore, disturbance history can have major effects on amount and connectivity of suitable habitat, as well as viability of populations (Amarasekare and Possingham 2001). Introducing simulation approaches that explicitly include habitat dynamics would further improve insights into dynamics of genetic diversity under variation in disturbance regimes.

In addition, the basic population processes of many species may not be static in response to disturbance regimes. For instance, the reproductive strategies of many plants are tied to disturbance (Gill 1975), and animal dispersal behavior and population connectivity may be variable in response to recent disturbance history (Templeton et al. 2001; Berry et al. 2005; Pereoglou et al. 2013). These responses will vary with the degree of phenotypic plasticity exhibited by the species in question (Anderson et al. 2012).

#### Disturbance modelling

Significant scope exists to model specific disturbance types more mechanistically using simulation methods that vary with respect to complexity, stochasticity and process representation (Bates and De Roo 2000; Keane et al. 2004; Seidl et al. 2011a). For instance, a consideration of dispersal distance of organisms suggests that disturbance shape, among other factors such as fire patchiness and minimum fire return interval, is also critically important given characteristics such as length‐to‐breadth ratio of disturbance events will influence the rapidity of recolonization dynamics and hence dynamics in genetic diversity.

#### Assumptions about genetic processes

There are two further areas where this study might be extended. Firstly, gene flow patterns are strongly tied to demographic processes in our model, but there are scenarios where gene flow can be decoupled from dispersal and colonization processes where, for example, animal dispersal is sex‐biased or plants show major differences in seed and pollen dispersal capabilities. Secondly, selection‐driven genetic responses to disturbance history have been documented (Moreira et al. 2014). Recent theoretical models have focussed on evolution under spatio‐temporal environmental heterogeneity (Blanquart and Gandon 2011), and empirical studies quantifying selective effects of disturbance regimes on traits and genes would be valuable for extending the present study.

## Conclusions

Our findings highlight the background conditions under which species must evolve strategies to persist in an environment of recurrent disturbance. Key to this are survival and dispersal strategies within the context of the temporal and spatial scale of patterns of disturbance regimes. Disturbance size, intensity, and frequency present conditions within which life‐history parameters, dispersal strategies, and density‐dependent behaviors may serve to limit or enhance genetic diversity within in situ residual populations and at the invasion front, as these are the basic mechanisms driving patterns of genetic diversity in an environment subject to recurrent disturbance.

## Conflict of Interest

None declared.

## Supporting information


**Appendix S1**. Additional quantification of the relative importance of seven other experimental treatments referred to in this article: landscape size; landscape topology; variable disturbance sizes; carrying capacity; negative exponential dispersal kernel; the number of alleles studied and the time during the annual cycle, when the population is limited to carrying capacity.

**Appendix S2**. Overview, Details and Design concepts (ODD) of the model used in this article following the suggested protocol by (Grimm et al. 2010).

**Appendix S3**. Java source code, parameter and input files together with installation instructions for the model used in article.
