# Supplementary material for: Implications of recurrent disturbance for genetic diversity
Source: Ecol Evol. 2016 Jan 25;6(4):1181–96. doi: 10.1002/ece3.1948 (PMC4725449; doi:10.1002/ece3.1948)
Supplement: Supplementary file 1 — Appendix S1. Additional quantification of the relative importance of seven other experimental treatments referred to in this article: landscape size; landscape topology; variable disturbance sizes; carrying capacity; negative exponential dispersal kernel; the number of alleles studied and the time during the annual cycle, when the population is limited to carrying capacity. [file ECE3-6-1181-s001.pdf]

Appendix S1. Supporting material

This appendix contains results from additional simulation experiments performed to explore the sensitivity of the GDDM model (Appendix S2) to seven experimental factors beyond those reported in the accompanying article. Factors and factor interactions are noted as *important* if they explain more than 2% of the variance in F_{ST} averaged over 1,000 generations preceded by a 4,000 generation burn-in period. Four replicates were used for these tests. Initial simulations were performed for 10,000 generations with 12 replicates to determine the burn-in time required to obtained reliable results (Figure 1).

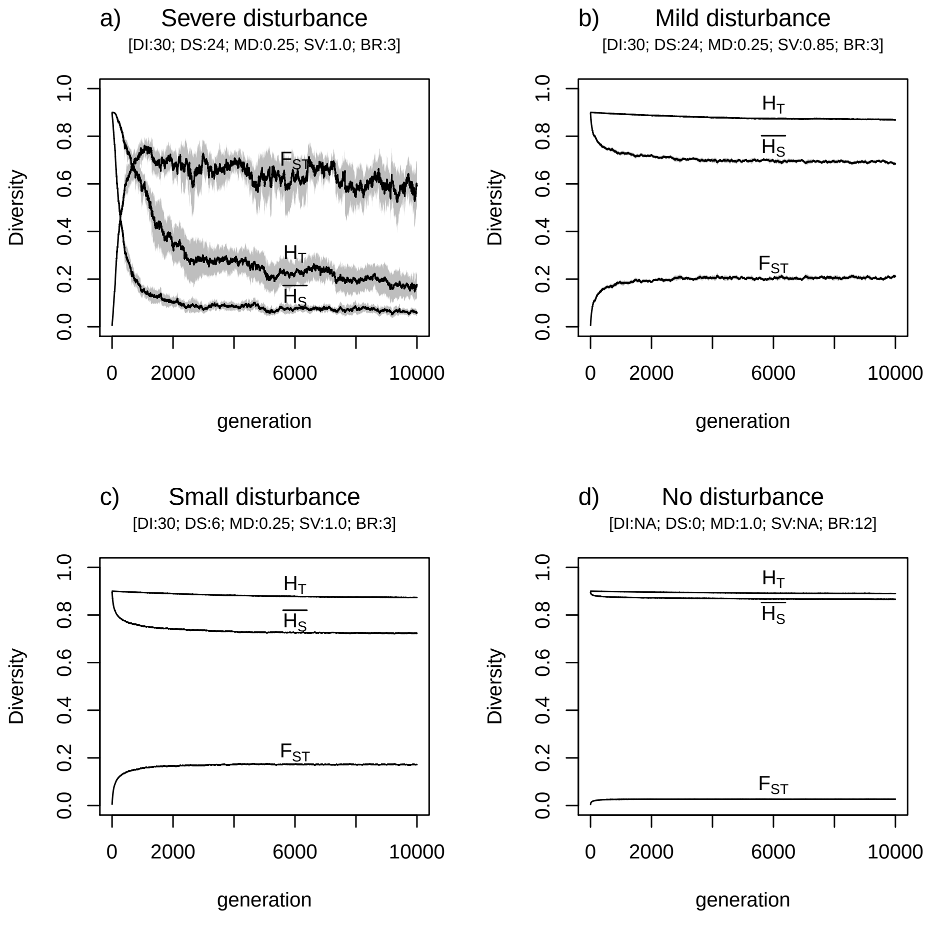

Figure 1: Time series of $H_T, \bar{H}_S$ and F_{ST}, with (a) and without (d) disturbance and two simulations with intermdiate disturbance values (b and c). Grey areas indicate the 95% confidence interval obtained from 12 replicate simulations.

An analysis of variance in F_{ST} was performed for each of the experiments and compared to that in the accompanying paper (Fig. 2.d). The nature of these differences is further explored through additional plots as required. The treatment levels are the same as those detailed in Table 1 in the accompanying article, unless otherwise indicated. Birth rate has not been included as an experimental factor because of the large number of simulations that would be required. For some experiments, (v, vi and vii below), disturbance size had to be decreased or disturbance frequency increased in order to maintain an extant population.

The seven factors are:

- i Carrying capacity (K). Treatment levels were 20, 40 or 80 individuals per cell;
- ii Dispersal method (DM). Treatment levels were *stepping-stone* or *negative exponential* dispersal kernals;
- iii Landscape size (LS). Treatment levels were 102 x 102 (Standard) or 204 x 204 cell landscape (Large);
- iv The number of alleles at each loci (NA). Treatment levels were 2, 10 or 50 alleles per locus;
- v Landscape topology (LT). Treatment levels were *Infinite* or *finite* with a surrounding buffer zone of 24 cells width excluded from analysis;,
- vi Disturbance size distribution (DSD). Treatment levels were constant or a log-size distribution; and,
- vii Time in the life-cycle when population is limited to K. Treatment levels were *before* or *after* dispersal.

Results

Carrying capacity (K) All single experimental factors became less important in explaining variance in F_{ST} as K became larger, with the exception of severity (SV) (Figure 2). In general, a reduced sensitive can be expected because genetic drift is proportional to population size (Figure 3). Nevertheless, a different response of SV to K arose due to the fact that the absolute number of survivors from disturbance is necessarily greater for larger K. Therefore, the sensitivity of SV to the treatment range will differ (Figure 3) and produce less variability in outputs (Figure 4). In addition, the need for longer simulations for larger populations also means that IBD patterns will take longer to evolve (Figure 5)

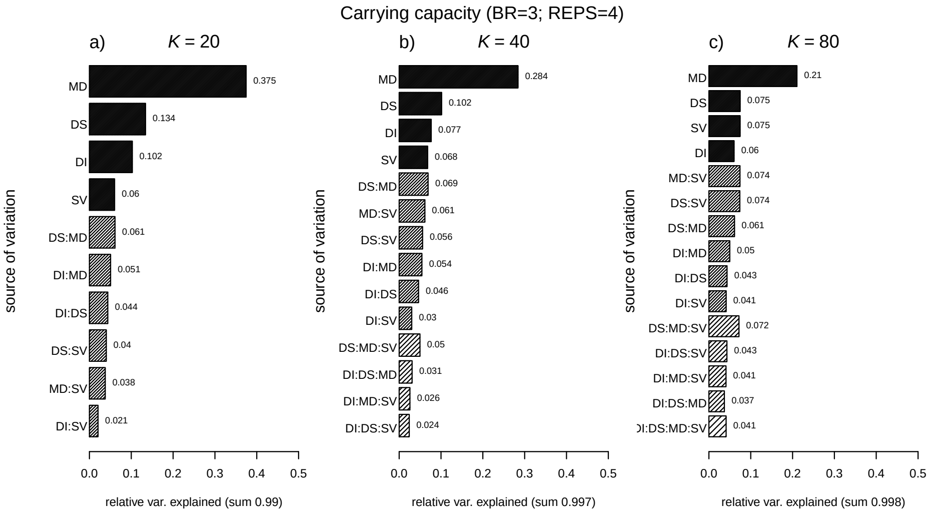

Figure 2: Variance in F_{ST} explained by disturbance size (DS), frequency (DI) and severity (SV) and mean dispersal distance (MD) for three different values of carrying capacity K.
Variance explained by SV is maintained as K becomes larger.

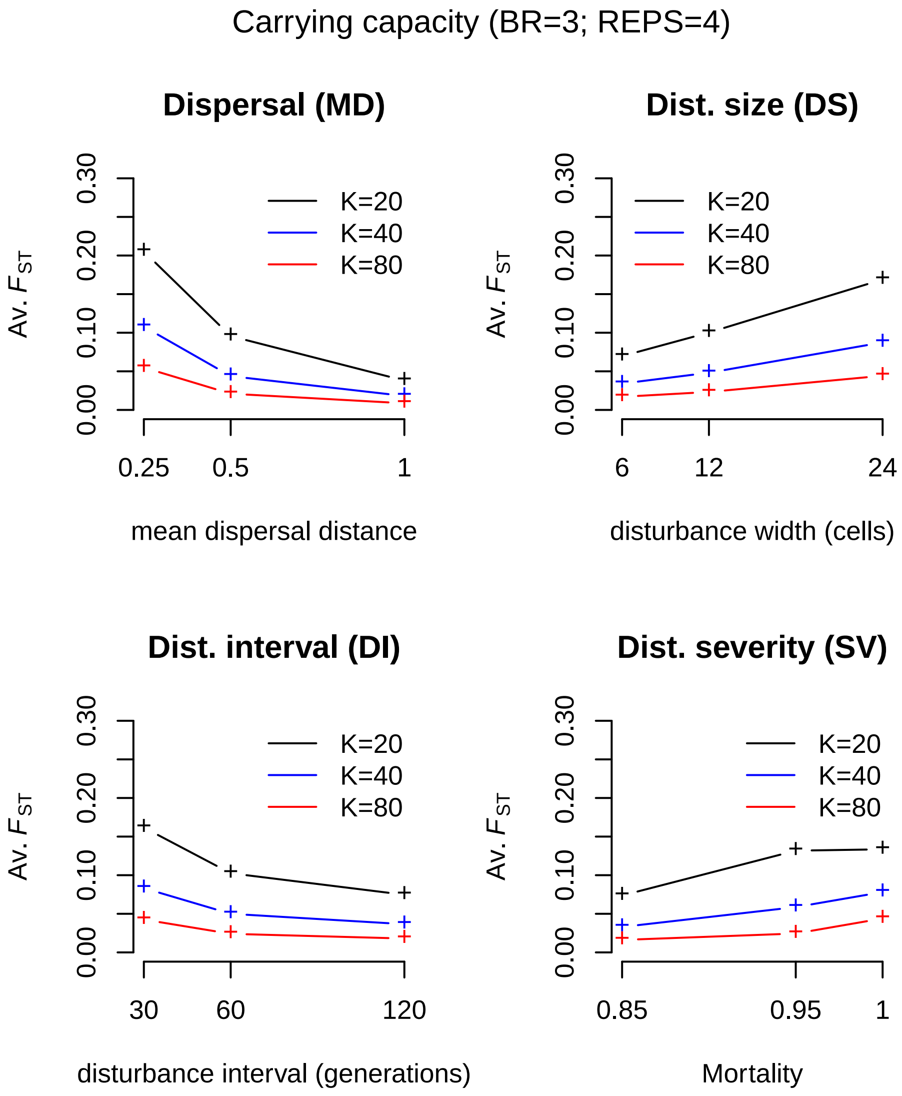

Figure 3: Trends in F_{ST} by four treatments for three values of carrying capacity *(K)*.
Larger populations produce a greater number of *in situ* survivors, leading to a different sensitivity range from the SV treatment.

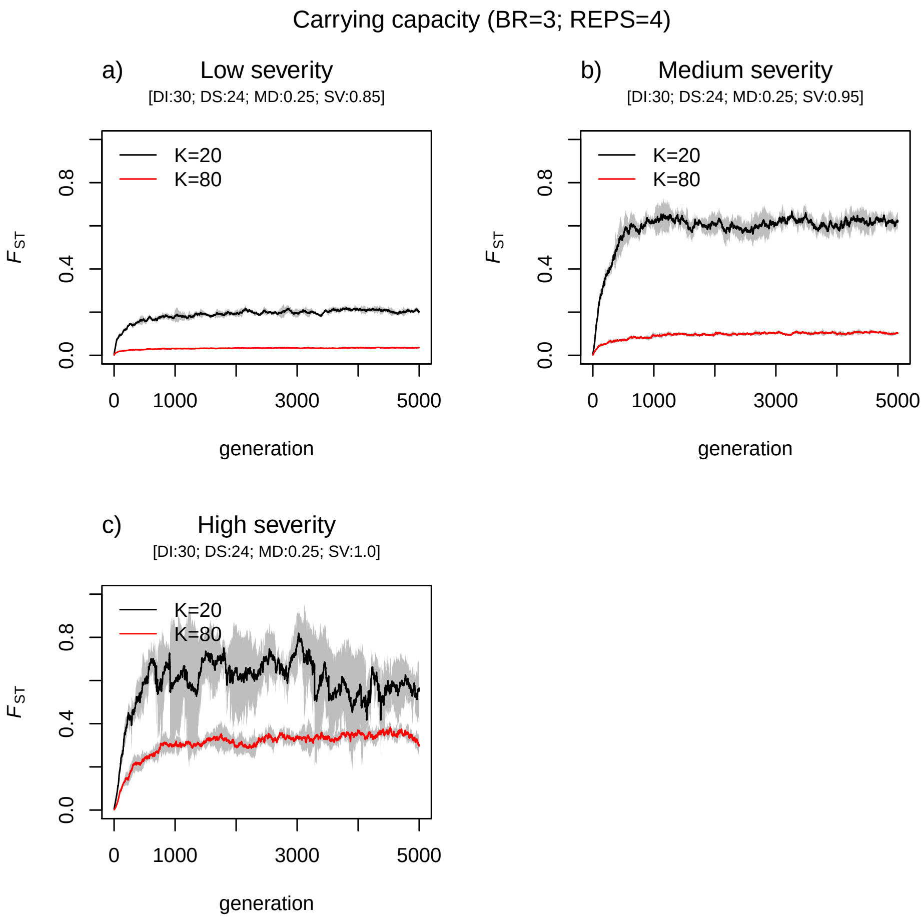

Figure 4: Time series of F_{ST} for three levels of minimum serverity.
Large populations produced less variability in F_{ST} and a greater absolute number of *in situ* survivors.

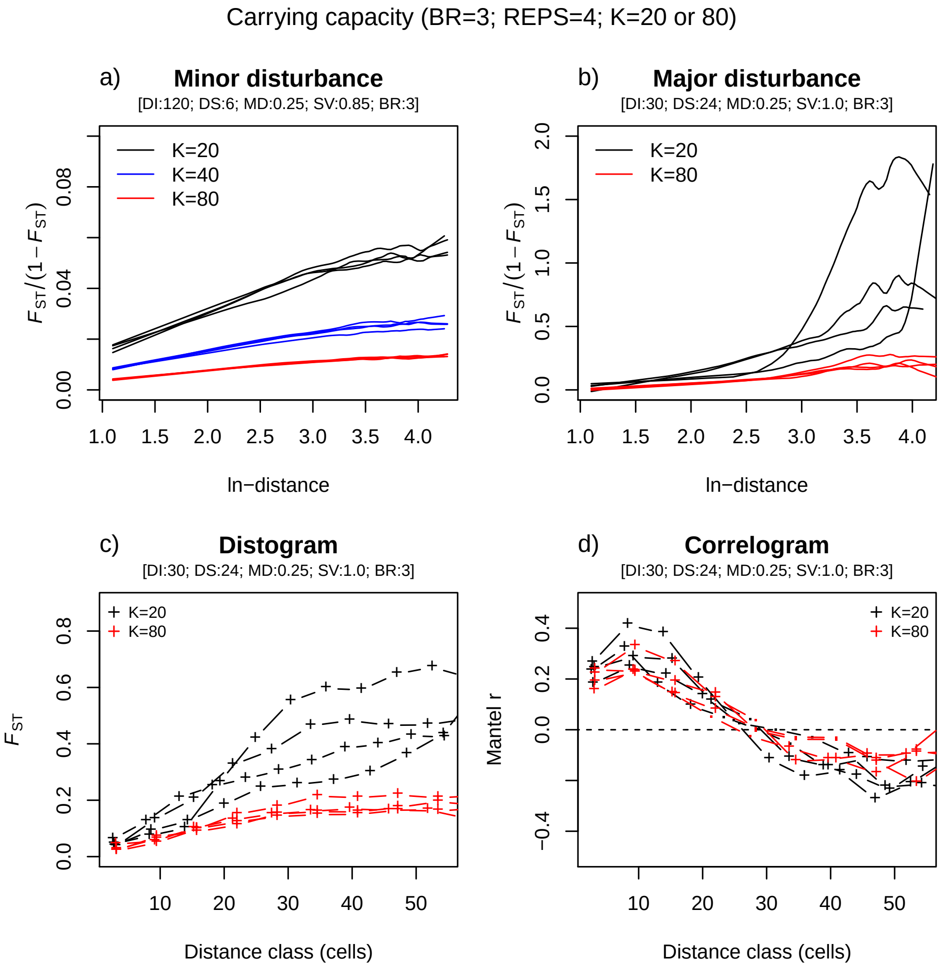

Figure 5: Pairwise F_{ST} for 50 randomly selected sites over four replicates.
For clarity, plots of pairwise transformed F_{ST} by *ln*-distance (a and b) are shown with Lowess regression lines (f=1/5). $K = 40$ has been omitted for charts (b) to (d). IBD patterns for larger populations have not developed to the same extent over 5,000 generations.

Landscape size (LS) Landscape size made little difference to variance explained in F_{ST} by experimental treatments (Figure 6) despite slightly higher sensitivity (Figure 7). IBD patterns indicate the landscape size used in the accompanying paper (*standard*), is possibly the smallest size that does not impede IBD patterns (Figure 8). Nevertheless, little can be concluded about *patch size*, the distance at which Mantel's r is zero, as it appears to scale to landscape size in this test (Figure 8.d).

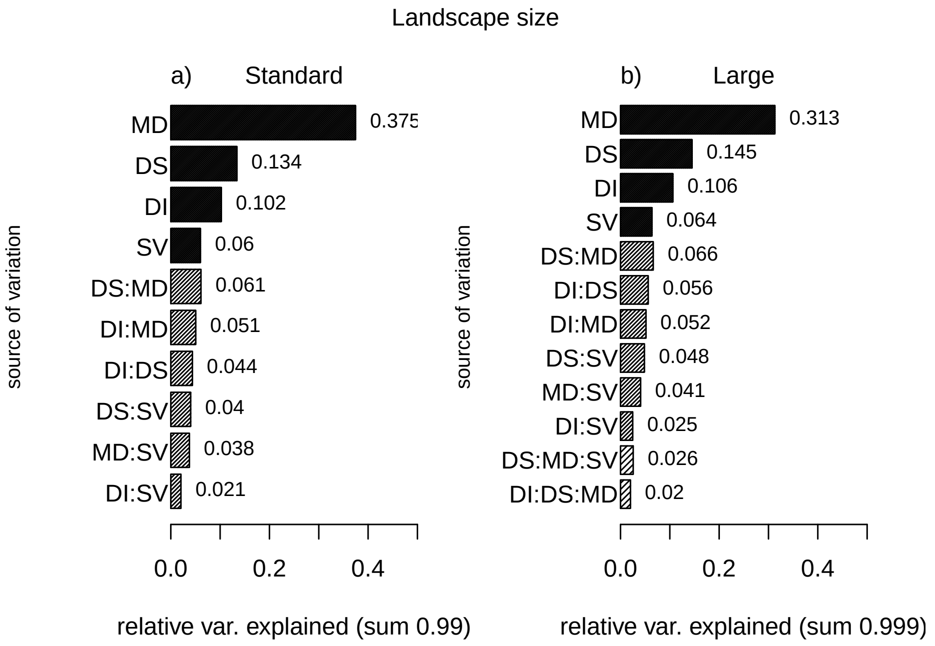

Figure 6: Variance in F_{ST} explained by disturbance size (DS), frequency (DI) and severity (SV) and mean dispersal distance (MD) for two landscape sizes.

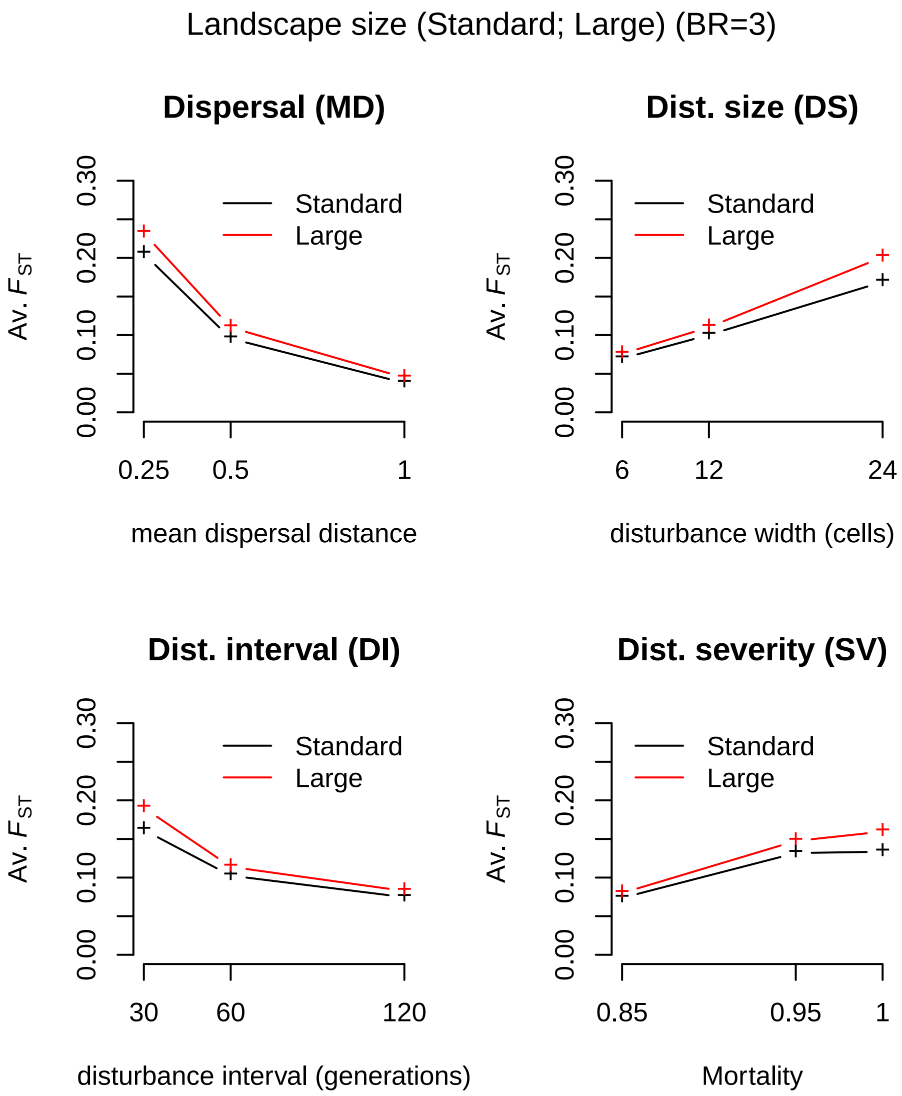

Figure 7: Trends in F_{ST} by four treatments for two landscape sizes.
The larger landscape produced slightly higher F_{ST} values for all treatments.

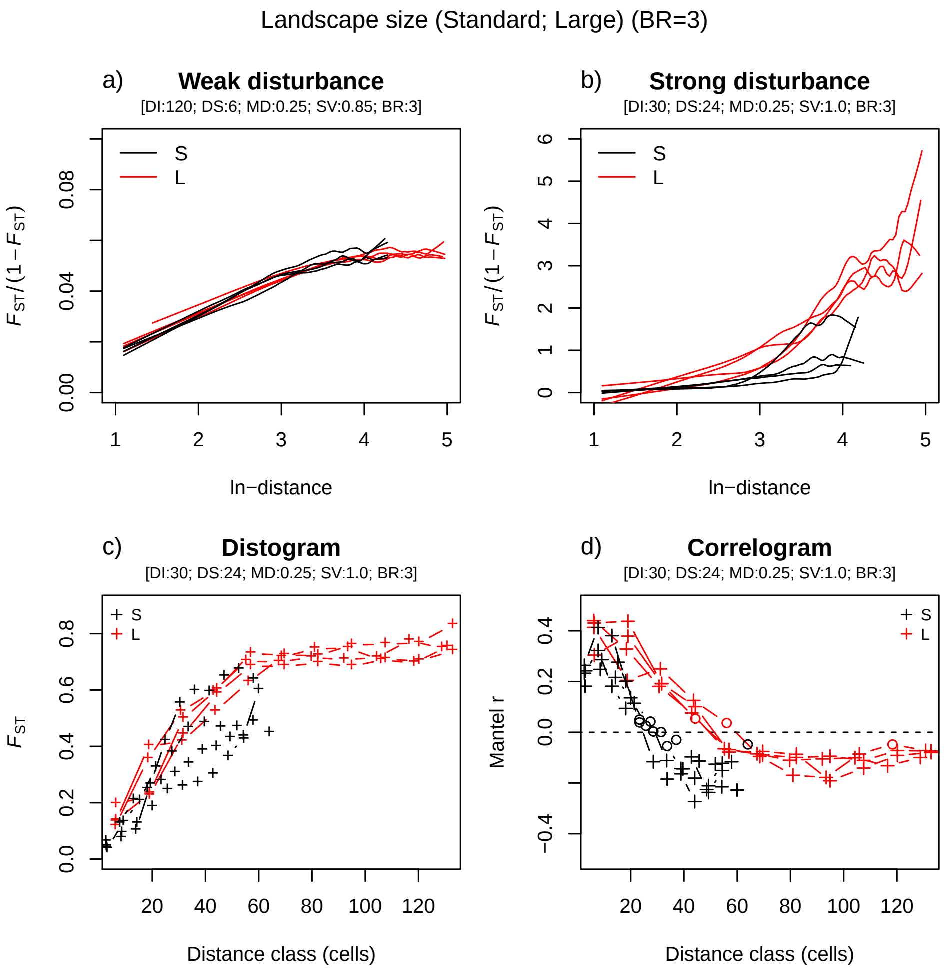

Figure 8: Pairwise F_{ST} for 50 randomly selected sites over four replicates for two landscape sizes.
For clarity, plots of pairwise transformed F_{ST} by *ln*-distance (a and b) are shown with Lowess regression lines (f= 1/5). Negative correlation is approximately constant after 70 cells. This is the maximum distance on a torus between any two cells for a landscape of 102 x 102 cells. However, patch size appears to scale with landscape size

Dispersal method (DM) The more realistic negative exponential dispersal model changed variance explained in F_{ST}, particularly for DI and SV (Figure 9,Figure 10). This difference is due to the larger number of migrant sources and the greater reach of dispersers which leads to more rapid recolonisation times.

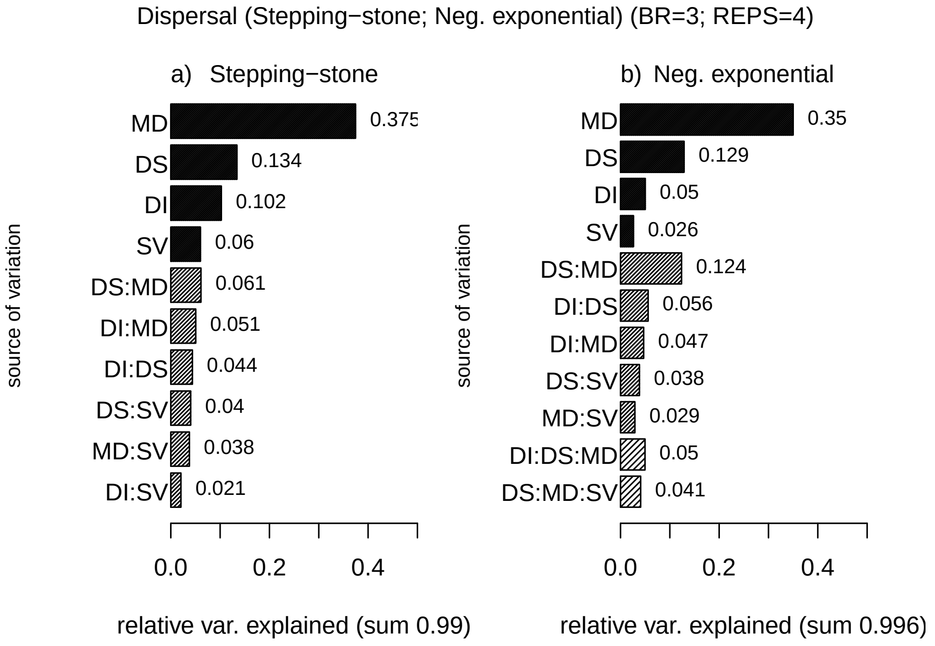

Figure 9: Variance in F_{ST} explained by disturbance size (DS), frequency (DI) and severity (SV) and mean dispersal distance (MD) for two different dispersal models.
The more realistic negative dispersal model reduces the importance of SV and DI.

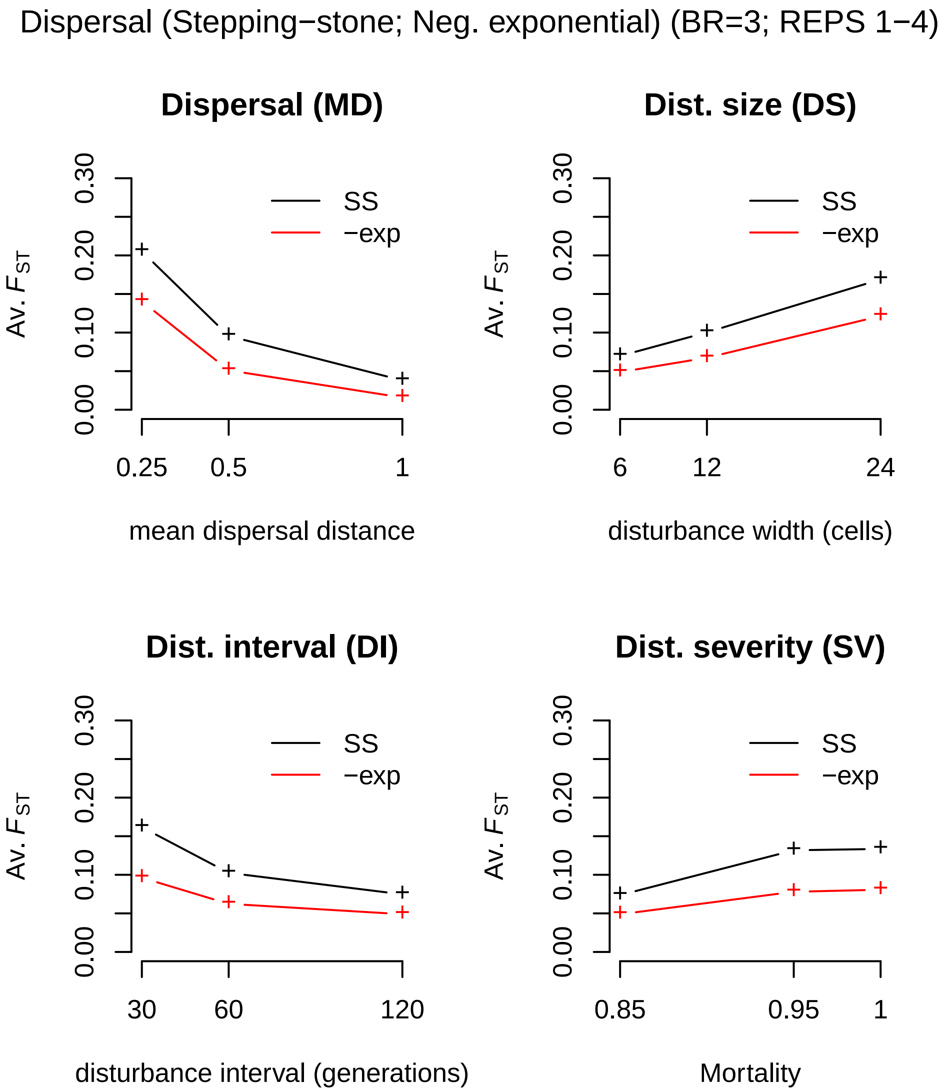

Figure 10: Trends in F_{ST} by four treatments for two dispersal models. The negative exponential model reduces F_{ST} for all experimental factors due to the large source of migrant sources and the short times required for recolonisation.

Disturbance size distribution (DSD) It might be expected that a log-size distribution of disturbances sizes will produce more variation in model outputs. However this is not manifest in the analysis of variance (Figure 11) but the trend is clear (if minor) for charts of trends in F_{ST} by treatment levels (Figure 12) and time series (Figure 13). IBD patterns are little changed (Figure 14). Note that to maintain extant populations for all scenarios, disturbance size (DS) treatments were 5, 10 or 20 cells, and disturbance interval was set to 160, 80 or 40 generations.

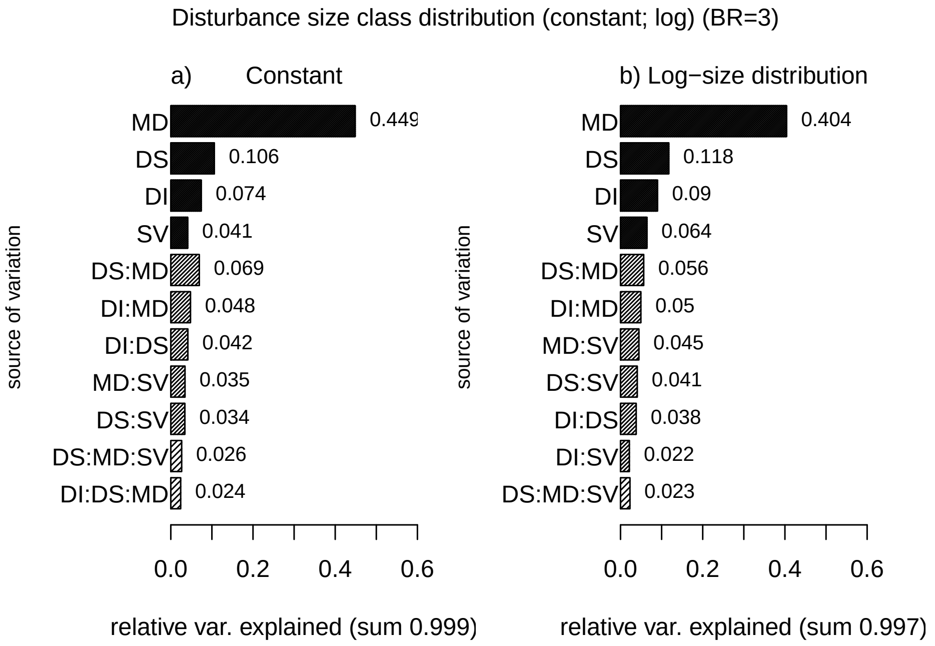

Figure 11: Variance in F_{ST} explained by disturbance size (DS), frequency (DI) and severity (SV) and mean dispersal distance (MD) for two different disturbance size-class distributions (*contant* and *log*).

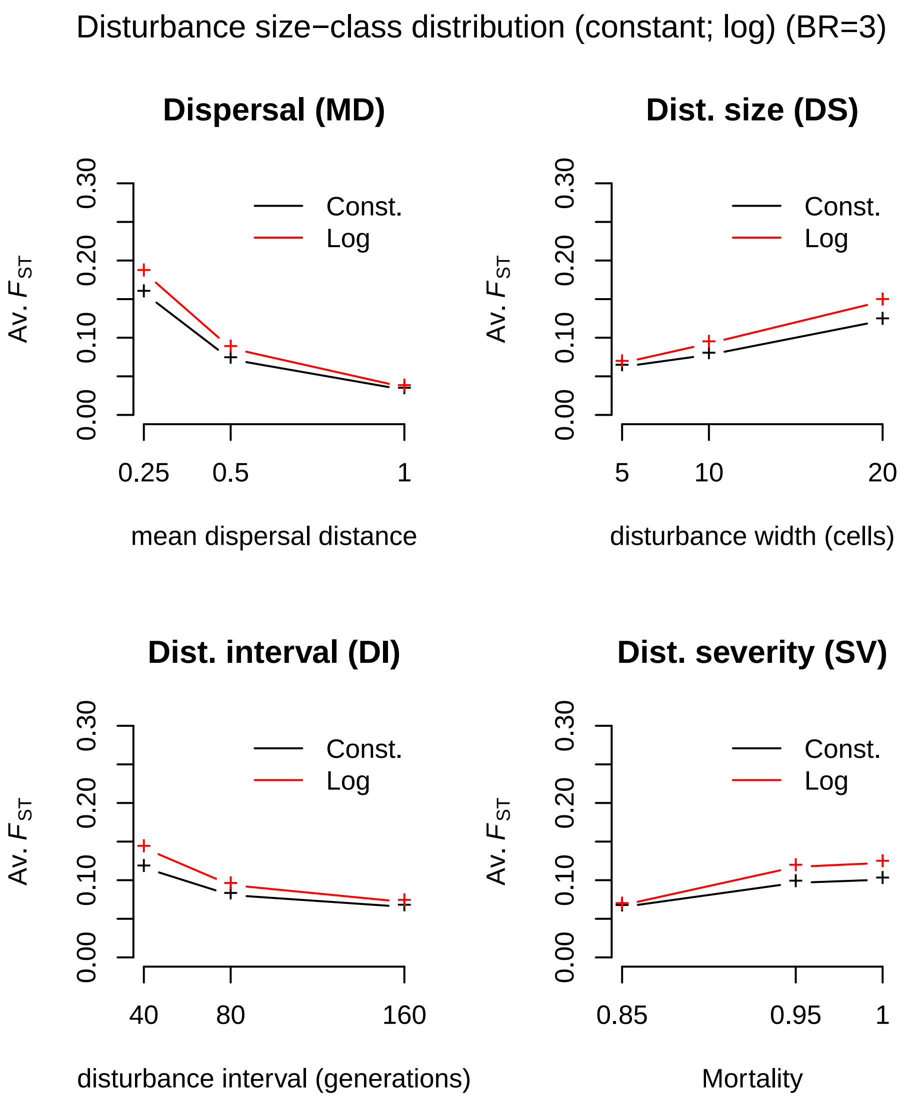

Figure 12: Trends in F_{ST} by four treatments for two disturbance size-class distributions (*contant* and *log*).
F_{ST} is greater for all treatment levels for the log-size distribution, indicating that large but rare disturbance sizes have a disproportional effect on F_{ST}.

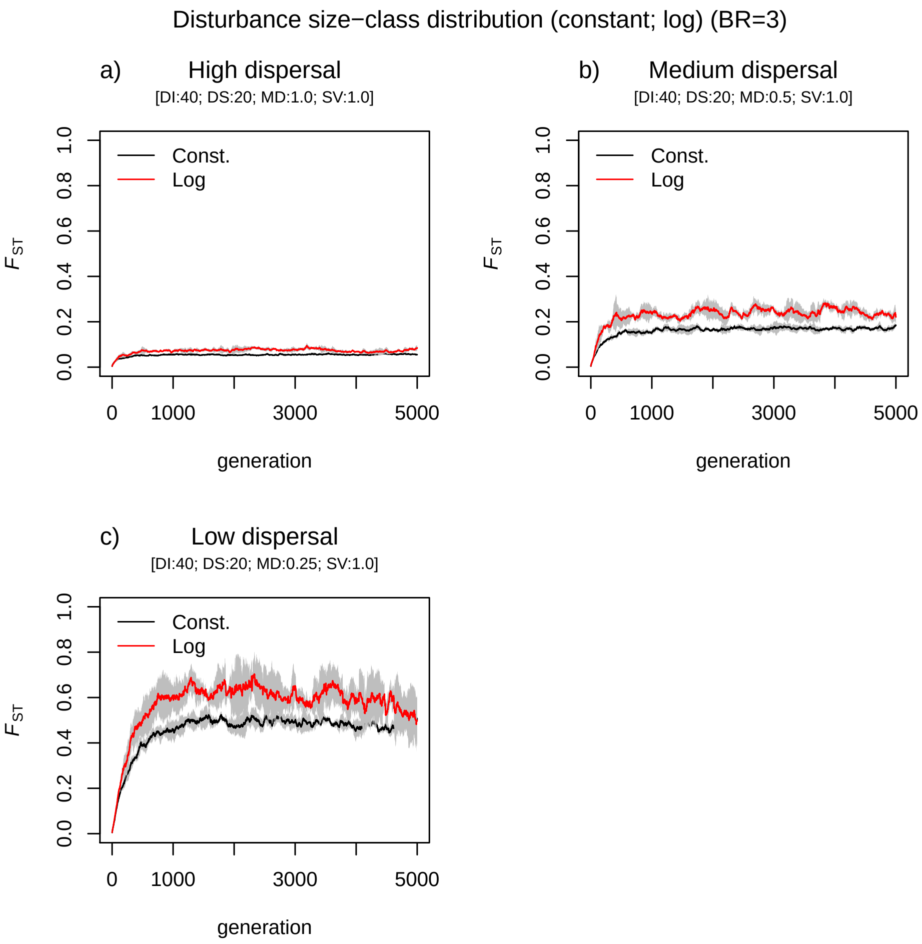

Figure 13: Time series of F_{ST} for three levels of minimum serverity and two disturbance-size distributions (*contant* and *log*).
The log-size distribution showed greater volatility in F_{ST}

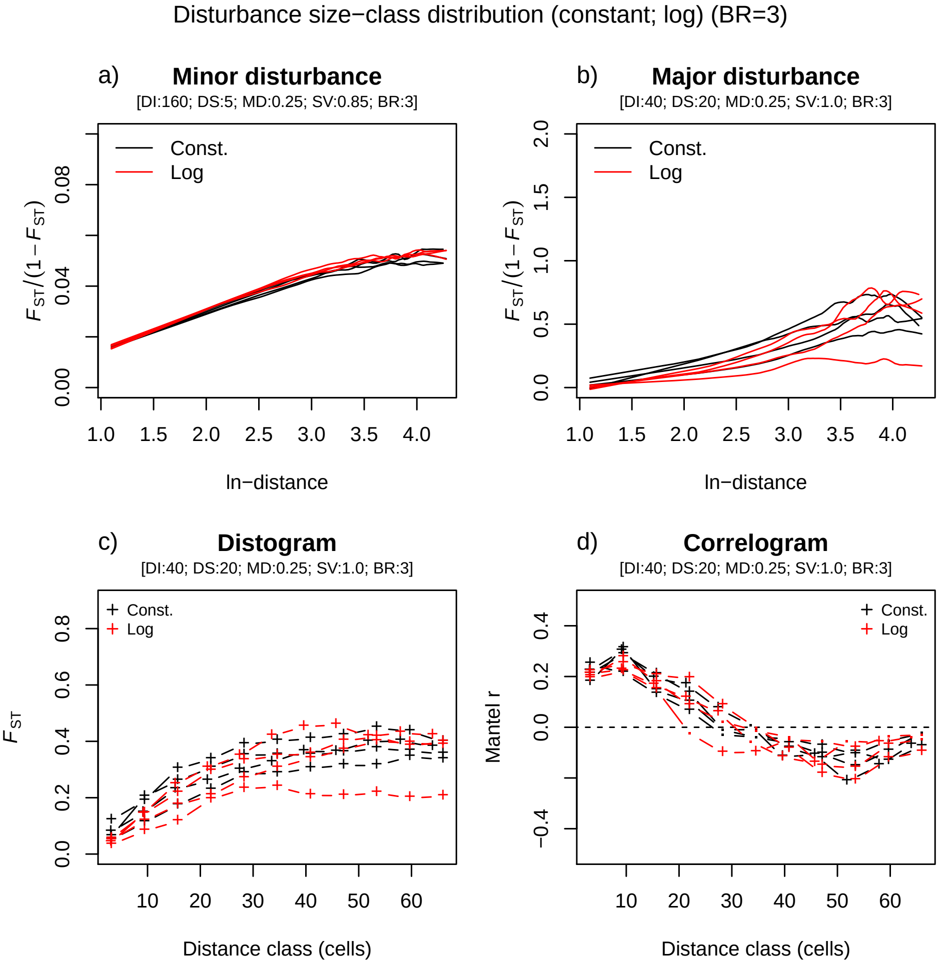

Figure 14: Pairwise F_{ST} for 50 randomly selected sites over four replicates. For clarity, plots of pairwise transformed F_{ST} by *ln*-distance (a and b) are shown with Lowess regression lines (f= 1/5).

Landscape topology (LT) Analysis of variance (Figure 15) and trends of F_{ST} over treatment levels (Figure 16) show little difference between the use of an infinite or finite landscape. Similarly, IBD patterns show little difference except for a change in patch size (Figure 17. A time series of the highly disturbed scenario for both topologies shows more volatility in F_{ST} for the finite landscape (Figure 18).

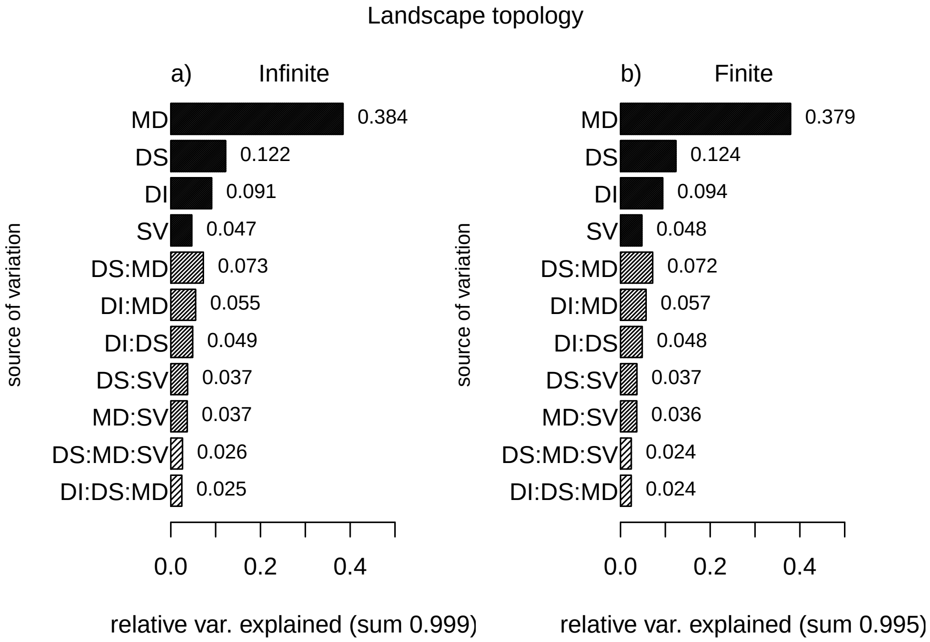

Figure 15: Variance in F_{ST} explained by disturbance size (DS), frequency (DI) and severity (SV) and mean dispersal distance (MD) for two different landscape topologies (*infinite* and *finite*).

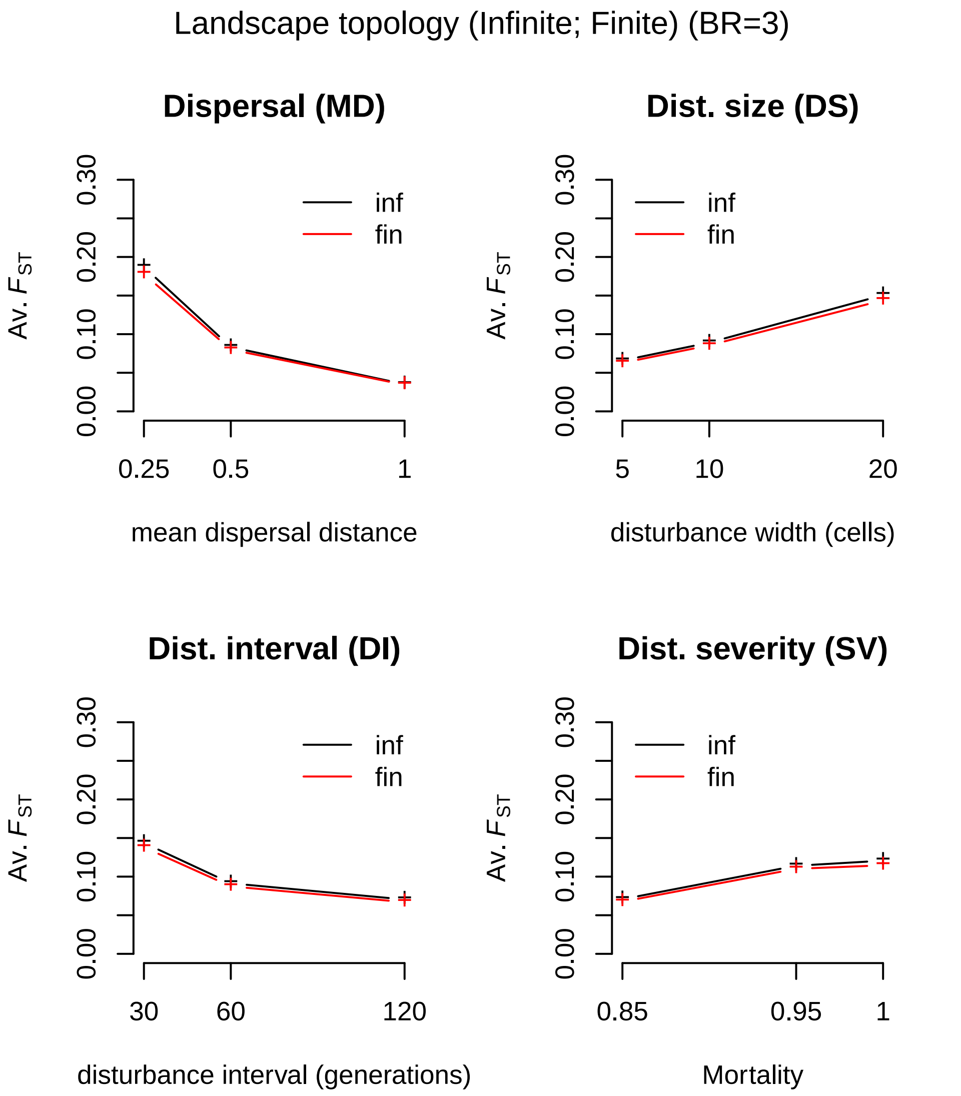

Figure 16: Trends in F_{ST} by four treatments for two landscape topologies (*infinite* and *finite*).

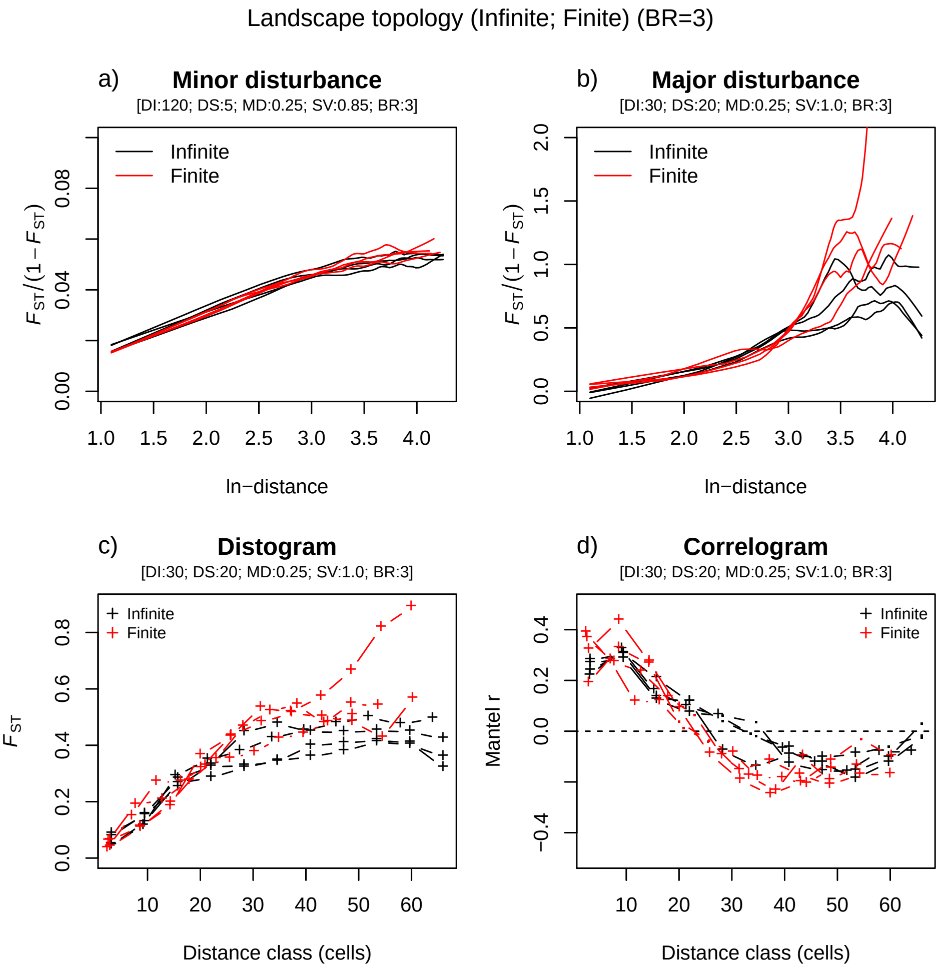

Figure 17: Pairwise F_{ST} for 50 randomly selected sites over four replicates for two differnet landscape topologies (*infinite* and *finite*).
For clarity, plots of pairwise transformed F_{ST} by *ln*-distance (a and b) are shown with Lowess regression lines (f= 1/5).

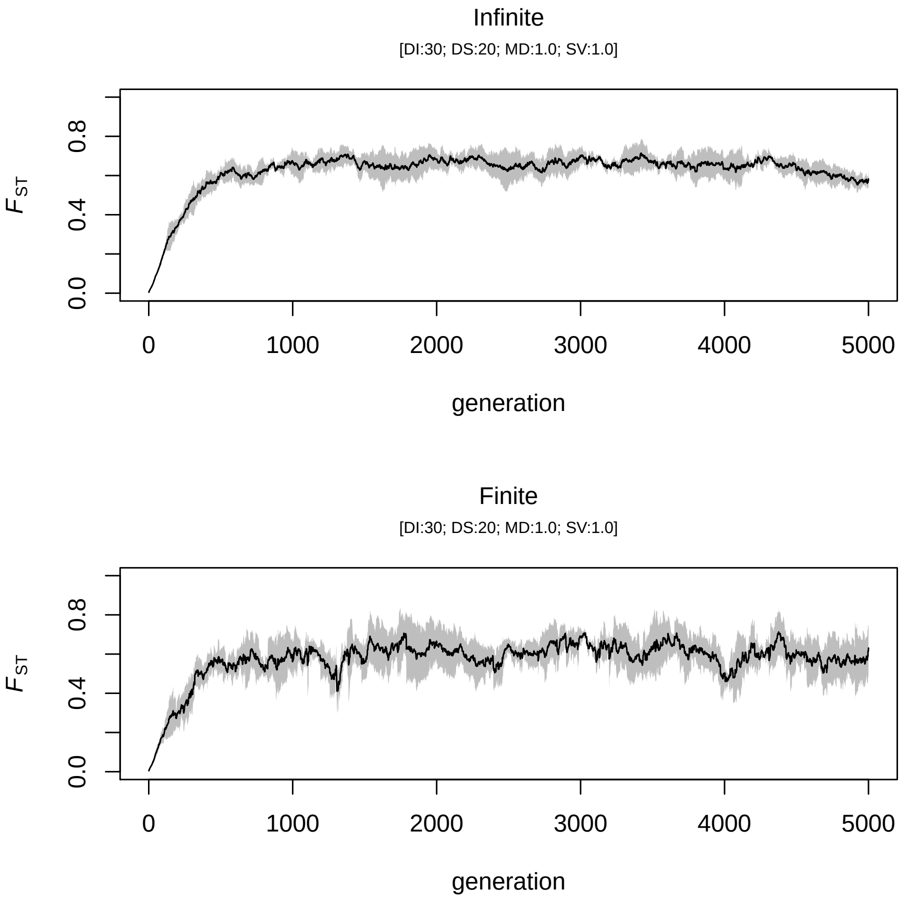

Figure 18: Time series of F_{ST} for two landscape topologies (*infinite* and *finite*). Outputs show a greater volatility for the finite landscape

Number of alleles (NA) The choice of the number of alleles did not manifest itself in differences of analysis of variance (Figure 19), trends in F_{ST} over treatment levels (not shown) nor IBD patterns. While the trends in IBD patterns were the same, the scatter of pairwise transformed F_{ST} values at each distance was proportional to the number of alleles used in the simulation for analysis (Figure 20).

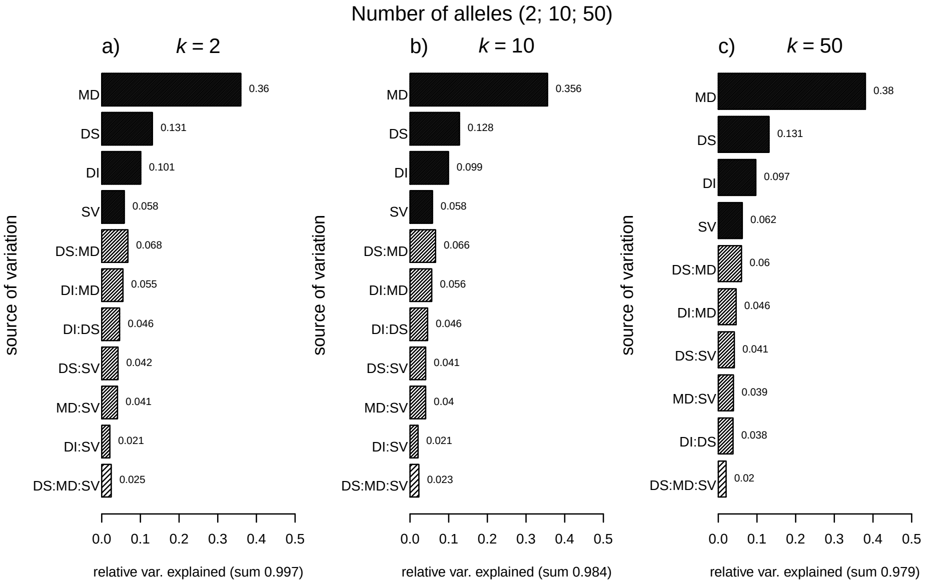

Figure 19: Variance in F_{ST} explained by disturbance size (DS), frequency (DI) and severity (SV) and mean dispersal distance (MD) for two different landscape topologies (*infinite* and *finite*).

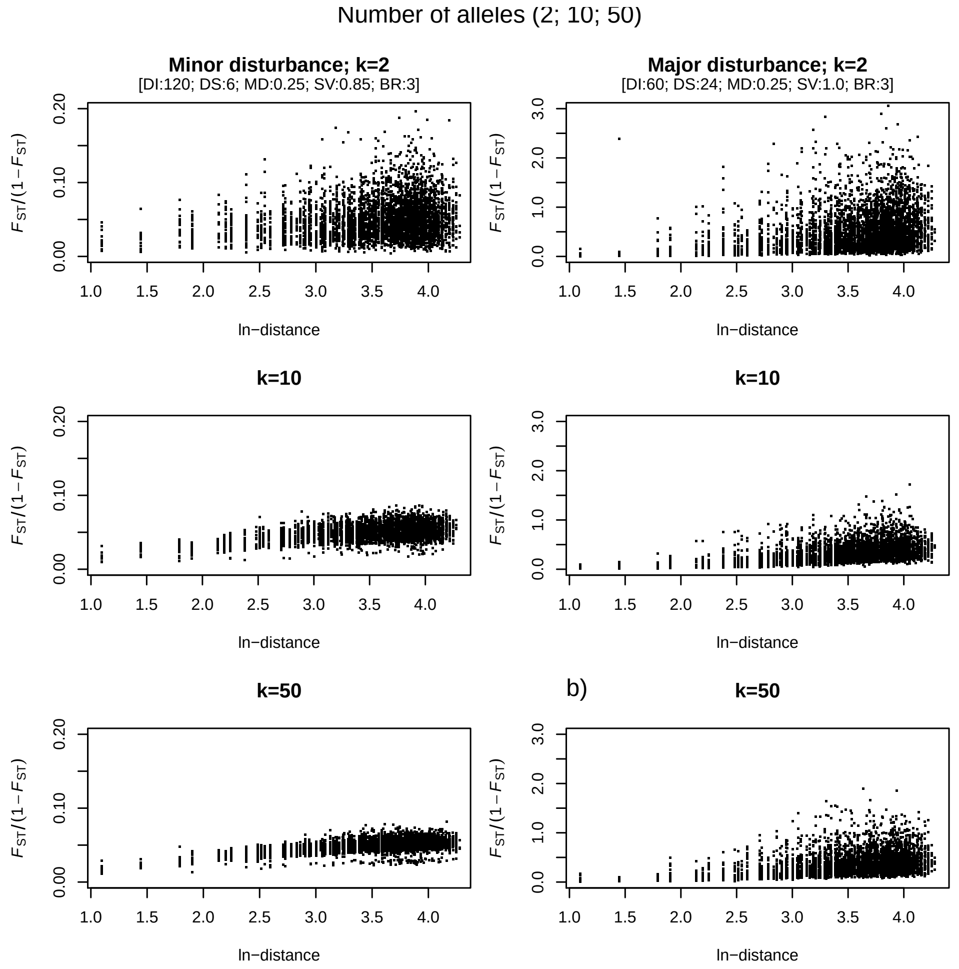

Figure 20: Pairwise transformed F_{ST} by log-transformed distance for 50 randomly selected sites over four replicates using three values for k in a k-allele model.

Time in the life-cycle when population is limited to K Limiting the population to K before, rather than after dispersal, reduces recolonisation rates. This has little effect on the relative importance of experimental treatments (Figure 21) but it could be expected that if birth rate had been a treatment, its effect would be substantially reduced. Because of reduced recolonisation rates F_{ST} is increased for all tested treatments when population is limited before dispersal (Figure 22. It follows that distruption to IBD patterns by disturbance will be greater for pre-dispersal population limiting (Figure 23).

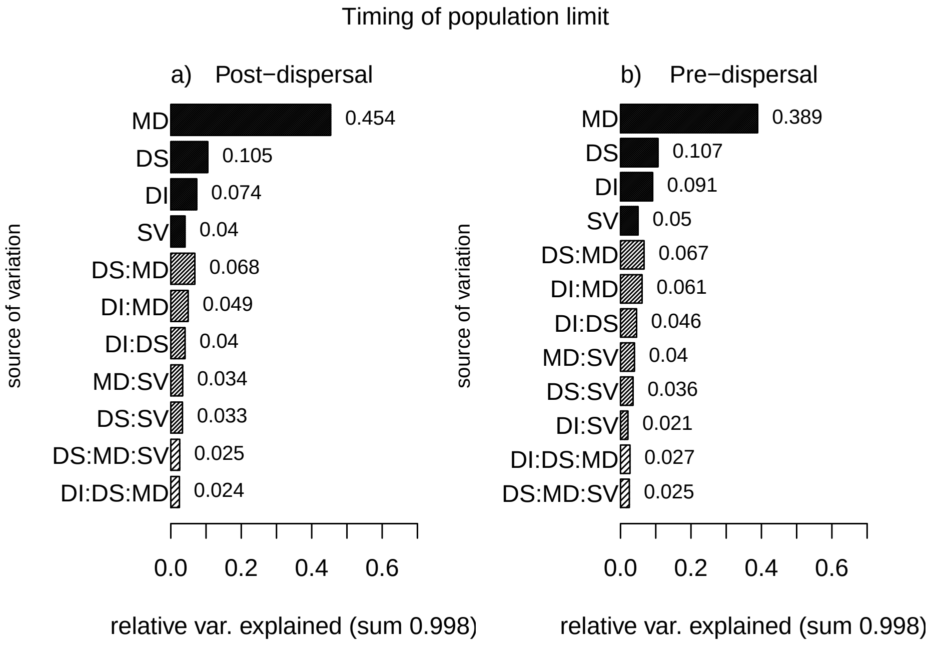

Figure 21: Variance in F_{ST} explained by disturbance size (DS), frequency (DI) and severity (SV) and mean dispersal distance (MD) when population limits are imposed before and after dispersal.

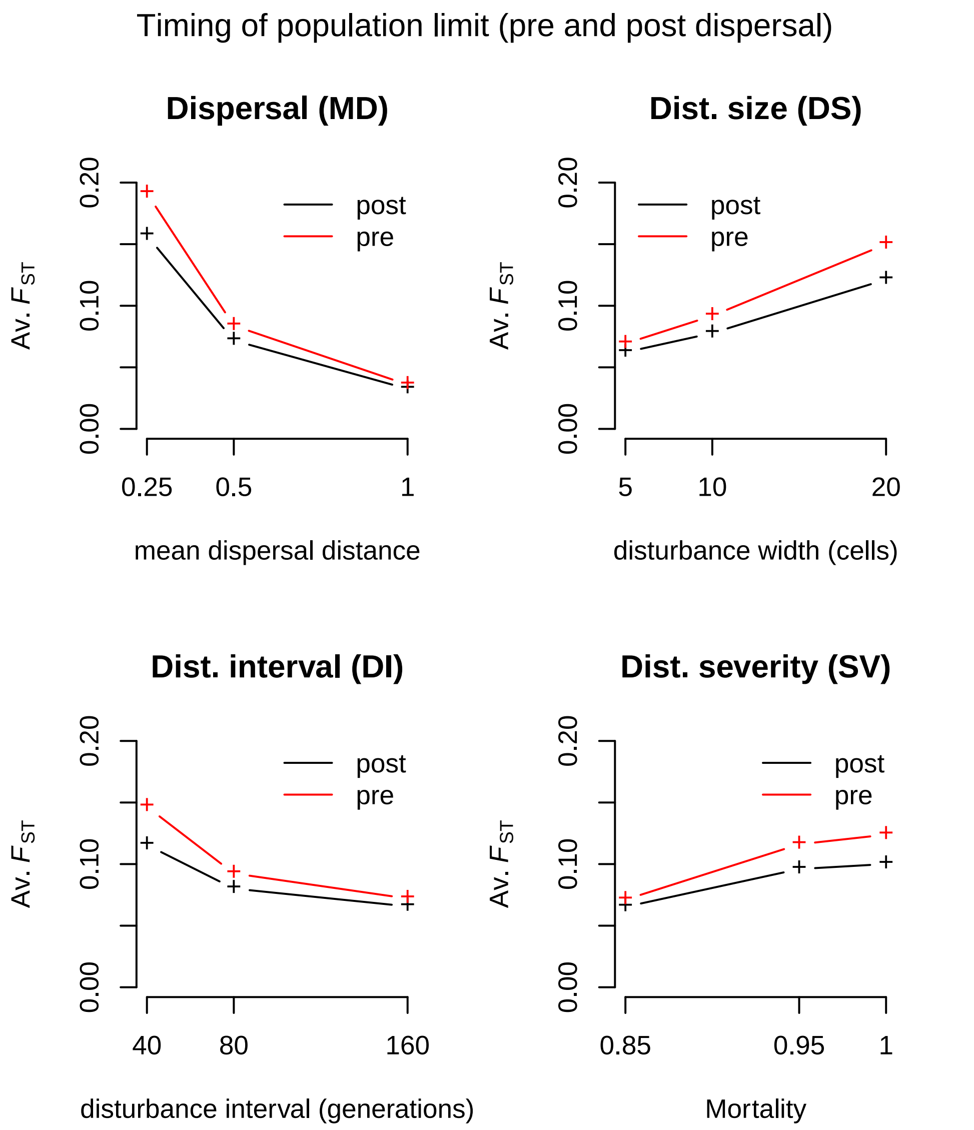

Figure 22: Trends in F_{ST} by four treatments for pre and post dispersal limitation of population to K.

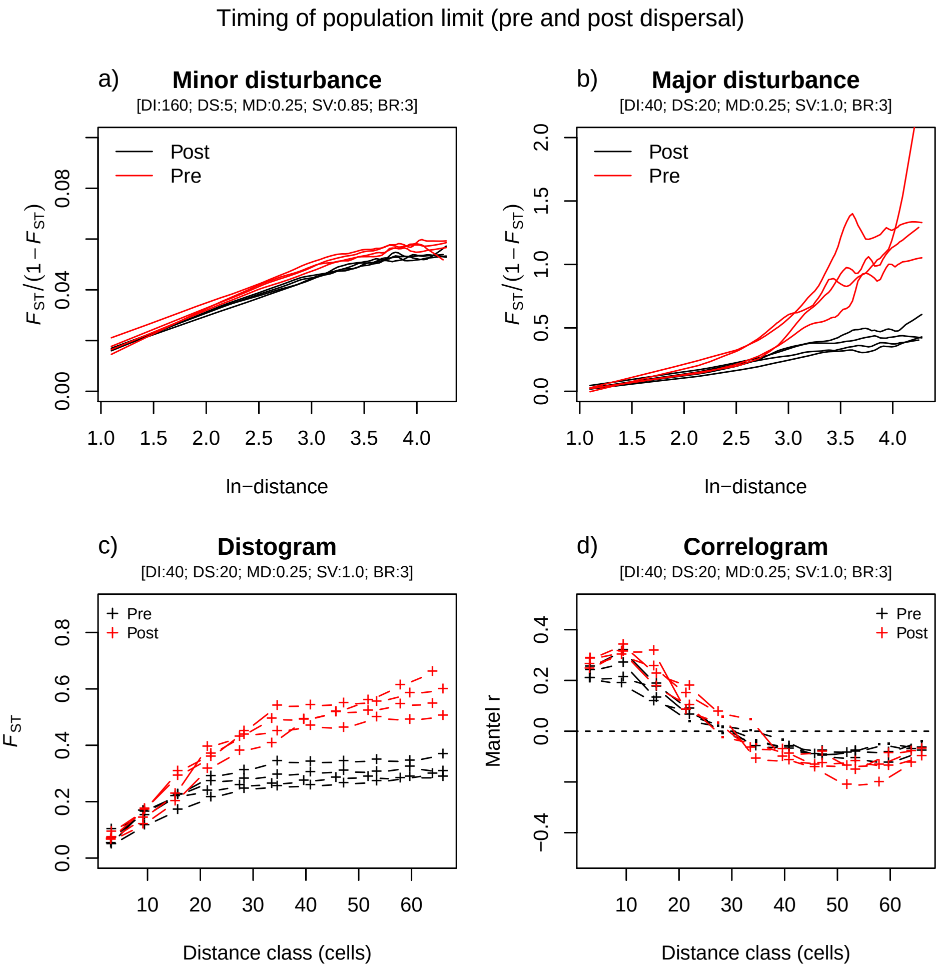

Figure 23: Pairwise F_{ST} for 50 randomly selected sites over four replicates for comparison of the timing when population limit is limited to K.
For clarity, plots of pairwise transformed F_{ST} by *ln*-distance (a and b) are shown with Lowess regression lines (f= 1/5).
