# Supplementary material for: Implications of recurrent disturbance for genetic diversity
Source: Ecol Evol. 2016 Jan 25;6(4):1181–96. doi: 10.1002/ece3.1948 (PMC4725449; doi:10.1002/ece3.1948)
Supplement: Supplementary file 2 — Appendix S2. Overview, Details and Design concepts (ODD) of the model used in this article following the suggested protocol by (Grimm et al. 2010). [file ECE3-6-1181-s002.pdf]

Appendix 2. Model Overview, Design concepts and Details

This model description follows the ODD (Overview, Design concepts, Details) protocol for describing individual- and agent-based models (Grimm *et al.* 2006, 2010) and is intended to be a self-contained document.

1 Purpose

The purpose of GDDM (Genetics, Demography and Disturbance Model) is to identify and rank the key drivers of genetic diversity for a single species under disturbance regimes in a neutral landscape. The model is intentionally simple and does not include cost surfaces for mate selection or dispersal as is common with many landscape genetics models. Two key sub-models: Dispersal and Disturbance, can be replaced by others as required.

GDDM is used to understand how patterns of genetic diversity within and between populations may vary in response to disturbance frequency and size and to ask how these may be influenced by simple variation in dispersal patterns, population growth rates and demographic structure.

2 Entities, state variables and scales

GDDM is a single species model containing two types of entities: individuals and cells. Individuals reside within a cell, are either male or female and belong to one of four age classes (new born, juvenile, adult or senescent).

There can be $0..n$ juvenile age classes, characterised by mortality (m_j) (i.e. mortality m for juvenile age class j per time step Δt) and $0..n$ adult age classes, characterised by birth rate (b_a) (i.e. birth rate b per female in age class a) and mortality (m_a). New born mortality is assumed to be included in the birth rate. Individuals remain in an age class for one generation until the final age class where they remain until removed by mortality. There must be one *New born* and one *Senescent* age class.

Genomes can be haploid or diploid, have one or more autosomal independent loci ($1..n$) and two or more alleles ($2..k$) at each locus. There is also a single mtDNA marker.

The simulation landscape is a grid of cells, each with a carrying capacity K and an x, y coordinate. Landscape processes (dispersal and disturbance) may view the landscape as either a torus (infinite) to avoid edge effects, or a finite bounded landscape.

3 Process overview and scheduling

Each time step (or generation) comprises processes listed Box 1.

Box 1. Scheduling pseudo-code

```
Initialise
  assign carrying capacity to each cell
  fill cells with random new born individuals*
  generate disturbance patterns for simulation*
for each generation
  for each cell
    age:  move all age classes to next age class
    mate:  randomly select mating pairs
      for each pair
        for each birth**
          for each locus
            select allele*
          create new born with selected alleles
          for each locus if mutate*
            select random allele from k-allele set*
          assign sex* (p < P_f)
    mortality:  remove individuals* (p < m_i)
    departure:  select eligible dispersers*
    for each dispersing individual
      determine distance*
      determine direction*
      round to cell coordinates**
      place in holding buffer
  arrival:  for each cell in buffer
    add arrivals to cell
  select disturbance pattern
  for each cell
    for each disturbance
      if disturbed
        apply disturbance mortality*
    remove individuals in excess of carrying capacity*
  observe:  if observation period
    select observation areas (N > N_min)
    for each observational area
      calculate H_S
    calculate F_ST, 'G_ST and D
select observation areas for pair-wise analysis*
```

(*) indicates stochastic elements

(**) indicates real numbers randomly rounded to integer values

4 Design concepts

Stochasticity All modelled processes include stochasticity, such as randomly assigned sex to new born individuals, mating pairs (with and without replacement), Mendelian inheritance, dispersal distance and direction, mortality and disturbance time and location (cell at the center of the disturbance shape).

Observation Within each cell resides a population ($0..K$). The heterozyosity (H_S) of this population is recorded during the simulation as is the population size, the total heterozygosity of the meta-population (H_T) and genetic difference (F_{ST}, $'G_{ST}$ and Jost's D). To ensure a sufficiently large sample size for analysis, the landscape can be divided into sets of cells, each comprising a square of $n.n$ neighbouring grid cells. The observer can view the landscape as a torus or a finite landscape with an edge buffer to exclude from observation. If the landscape is interpreted as infinite, distances between observational areas (for the purpose of pair-wise analysis) are the minimum distance between two points on a torus.

For a given population, heterozygosity is averaged over the number of loci l for frequency p of allele i.

$$H = \frac{1 - \sum_{i=1}^{k} p_i^2}{l} \tag{1}$$

The average heterozygosity of populations $\bar{H_S}$ is the sum of H_i weighted by population size N_i.

$$\bar{H_S} = \frac{\sum_{i=1}^{s} H_i N_i}{N} \tag{2}$$

Three measures of genetic difference between populations are provided:

$$F_{ST} = \frac{H_T - \bar{H_S}}{H_T} \tag{3}$$

$$D = \left(\frac{H_T - \bar{H_S}}{1 - \bar{H_S}}\right)\left(\frac{n}{n-1}\right) \tag{4}$$

$$'G_{ST} = \frac{\ln((1 - H_T)/(1 - \bar{H_S}))}{\ln(1 - H_T)} \tag{5}$$

This data, together with a running average of these outputs and their coefficients of variation, are produced continuously during the observational period of the simulation. Normally, a *'spin-up'* or *'burn-in'* period is allowed for before observations are recorded.

Genetic isolation-by-distance between populations A and B is measured as:

$$F_{ST}(AB) = \frac{H_T - (H_A N_A + H_B N_B)/N_A N_B}{H_T} \tag{6}$$

Distance is the Euclidean distance between A and B in cell units. This data is saved to file at the end of every simulation for a specified number of randomly selected cells. Cells with populations $< 0.1K$ are not included in any measure of heterozygosity.

In addition to the above data, maps are produced during simulation of H, N, Most frequent allele, mtDNA, disturbance and mean disturbance interval.

Emergence What emerges from the simulations is the heterozygosity of a dispersing population in the context of a disturbance regime (Figure 1).

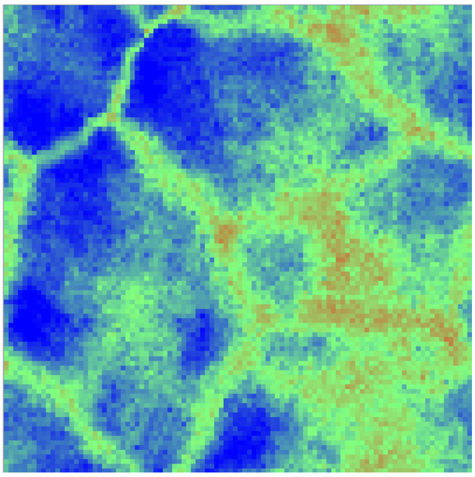

Figure 1: A snapshot of patterns of heterozygosity emerging during a simulation of a single species persisting in a homogeneous landscape experiencing recurrent disturbance

Adaptation The model is neutral with regard to any measure of fitness. In addition, birth and mortality are genetically non-selective.

Collectives The only entity which may be considered a collective is the population within a cell. These populations experience stochastic extinction at low population levels.

5 Initialization

Each cell is populated to the limit of the carrying capacity (K). All individuals have a random genome determined by the number of loci and the number of alleles available at each locus. Sex is randomly assigned in the

proportion of the probability of being female (P_f). Disturbances are exogenous to the model and their frequency and extent are calculated at this time.

6 Input data

K is read from an ArcGIS export file (*.asc) which also specifies the dimensions of the landscape. The dynamics of GDDM are driven by disturbances, dispersal and Mendelian genetics. The model assumes no other external environmental variation.

Input data also includes model parameters as well as the selection of dispersal and disturbance sub-models (tables 1 through 8)

Table 1: Temporal and spatial extents

Name	Range	Description
nSteps	[1..*any*]	one breeding cycle per time step
gx, gy	[1..*any*]	landscape dimensions in cell units

Table 2: Genetics

Name	Range	Description
nAlleles[]	[1..*any*]	array (dim=nLoci) elements are the number of alleles at each locus
haploid	$true/false$	hapoid or dipoliod genome
mutationRate	[0.0..1.0]	mutation rate per allele per birth

Table 3: Demography

Name	Range	Description
nJAC	$[0..any]$	number of juvenile age classes (not including new born)
nAAC	$[0..any]$	number of adult age classes (not including senescent)
birthRate[]	$[0.0..any]$	birth rate for each adult and the senescent age class per female per generation. Dim = $[1..nAC + 1]$
deathRate[]	[0.0..1.0]	death rate for each age class excluding new born. Dim = $[1..nJAC + nAAC + 1]$
pFemale	[0.0..1.0]	probability a new born is female
matingMode	[1..4]	(1) both with replacement; (2) female with replacement; (3) male with replacement; (4) both without replacement

Table 4: Disturbance (constant size)

Name	Range	Description
size	$[0..min(gx, gy)]$	width of square distances
minMortality	[0.0..1.0]	mortality varies randomly between this value and 1.0
idi	$[1..any]$	width of square disturbances
torus	$true/false$	Allow disturbance area to wrap around landscape edges

Table 5: Disturbance (log size distribution)

Name	Range	Description
meanSize	$[0..min(gx, gy)]$	mean width of square distances in a log distribution
minMortality	[0.0..1.0]	mortality varies randomly between this value and 1.0
idi	$[1.0..any]$	width of square disturbances
torus	$true/false$	Allow disturbance area to wrap around landscape edges

Table 6: Dispersal (Bath)

Name	Range	Description
meanDistance	$[0.0..min(gx, gy)]$	$d = m(2\epsilon - 1)$
torus	$true/false$	Allow individuals to disperse around landscape edges

Table 7: Dispersal (exponential)

Name	Range	Description
meanDistance	$[0.0..min(gx, gy).0]$	$d = -m \log(1 - p)$
torus	$true/false$	Allow individuals to disperse around landscape edges

Table 8: Observer

Name	Range	Description
torus	$true/false$	Interpret distances between population as a torus
guard	$[0.. < min(gx, gy)/2 - 1]$	Buffer width to exclude from observations
width	$[1..min(gx, gy)]$ $(\|gx \mod w = 0$ and $gy \mod w = 0)$	Width of number of cells in a square observation area
minSample	$1..K$	Minimum N for an observation area to be included in outputs
observeTime	$[1..nSteps]$	Time to begin observations
bufferSize	$[1..nSteps]$	Number of generations to include in running averages

7 Submodels

Aging All individuals move up one age class except the senescent age class.

Mating Mating pairs are selected using a choice of four algorithms: (1) both without replacement; (2) females with replacement; (3) males with replacement; and (4) both with replacement. Pairs are selected from the population within the cell. The size of population at $t+1$ is:

$$N_{(t+1)} = [N_{(f,t)} 2\lambda + Di - De - Dm] \qquad \{limitK\} \tag{7}$$

where:
$N(f,t)$ = number of females at time t;
λ = density-independent population growth rate;
D_i and D_e = the number of immigrants and emigrants respectively; and
D_m is the additional mortality due to disturbance.

Mortality An individual is removed from the population if a randomly drawn number from a uniform distribution is less than the mortality rate for the age class to which it belongs: $M_{ac} < \epsilon$. For non-overlapping generations, there are two age classes, new born and senescent with mortality = 1.0. This function is not applied to new born individuals, as it is assumed their mortality is incorporated in the birth rate.

Dispersal There are two dispersal algorithms to choose from, bath dispersal (Figure 2) and a negative exponential dispersal (Figure 3). For the bath dispersal, there is an equal probability of entering any cell within the radius of the dispersal distance.

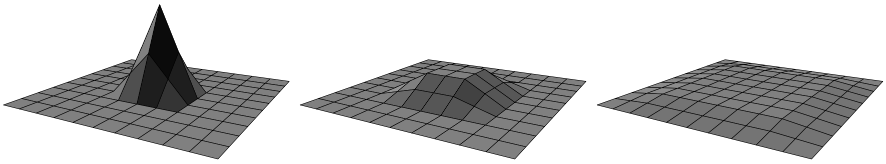

(a) $m = 1.0$ (b) $m = 2.0$ (c) $m = 4.0$

Figure 2: Examples of Bath dispersal
The lattice shown above is the value at the centroid of each cell (i.e. this is a lattice, the duel of the grid of cells.

If a cell is not entirely within the dispersal radius, the probability is area weighted. The coordinates of the destination cell are integer values. These are found by random rounding the coordinates from $\mathbb{R}^2$ to $\mathbb{I}^2$.

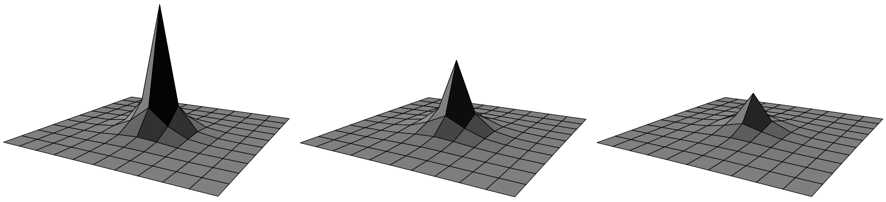

(a) $m = 1.0$ (b) $m = 2.0$ (c) $m = 4.0$

Figure 3: Examples of negative exponential dispersal
As in the previous figure, the lattice is the value at the centroid of each cell.

Disturbance There are two disturbance algorithms to choose from. Each of these algorithms produce an average inter-disturbance interval as specified by the '*idi*' parameter (Table 4). Both algorithms produce square disturbances, centered at a randomly selected cell and at a random time. The *constantDisturbance* produces disturbances all of the same size as specified by the *'size'* parameter (Figure 4.a). The *logDisturbance* sub-model produces disturbances with a log size distribution, *'meanWidth'* in size (Figure 4.b).

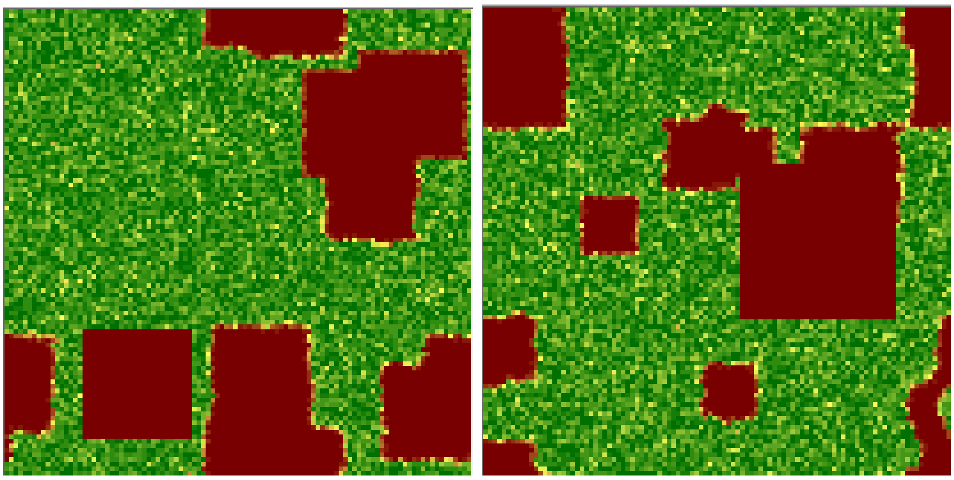

(a) constant size (b) log size distribution

Figure 4: A snapshot of simulatied population showing disturbance patterns
Simulated landscapes showing population size (N) (Green $N = K$, Brown ($N = 0$).

Bibliography
