# Supplementary material for: Implications of recurrent disturbance for genetic diversity
Source: Ecol Evol. 2016 Jan 25;6(4):1181–96. doi: 10.1002/ece3.1948 (PMC4725449; doi:10.1002/ece3.1948)
Supplement: Supplementary file 3 — Appendix S3. Java source code, parameter and input files together with installation instructions for the model used in article. [file ECE3-6-1181-s003.zip › Readme.rtf]

Setting up GDDM
This page tells you how to setup an Eclipse based development environment for the 3Worlds project in which the GDDM model is implemented.
Supported Platforms
These instruction apply to the use of Eclipse and associated tools on Linux. Setting up an OS-X and Windows environment is similar. Make sure the latest version of the Java Development kit is installed from Oracle. In Ubuntu, do not use the Open JDK that is installed by default.
Setting up Eclipse
Eclipse IDE
1.	Download the Eclipse IDE for Java Developers. (https://eclipse.org/downloads/)
2.	Start eclipse 
Eclipse plugins
Generally, you will only need the IvyDE plugin.
IvyDE plugin (http://ant.apache.org/ivy/ivyde/)
From Eclipse
1.	Select Help -> Install New Software ...
2.	Enter the following URL in the Work with field:
http://www.apache.org/dist/ant/ivyde/updatesite
3.	Click Add
4.	Click Next
5.	Wait until a list of products appears in the name list
6.	Select (check box) Apache Ivy library
7.	Select (check box) Apache IvyDE Eclipse plugins
8.	Click Next
9.	Click Next again
10.	Accept the license terms
11.	Click Finish
12.	Click OK on the Security Warning dialog
13.	Click Restart Now to restart Eclipse
Setting up an Eclipse project - Stage I
1.	Start eclipse in a workspace in which you would like to create your new project (Documents/workspace)
2.	File -> New -> Java Project
o	Enter the name “threeWorlds” in the project name field
3.	Click 'Finish'
4.	Right-click the 'src' directory and select 'Delete'
5.	Click 'OK to confirm
6.	From the main menu, select Eclipse -> Quit Eclipse
7.	Click OK to exit Eclipse
Getting the source code:
The zip file (AppendixS3) contains two archives: .3w.zip and src.zip. Extract src.zip to the threeWorlds directory created in the previous step. 
Extract .3w.zip to you home directory – the root directory of your user name. These are the parameter and configuration files for GDDM.
Extract the ivy.xml file to 'threeWorlds'
Setting up an Eclipse project - Stage II
Start Eclipse and refresh your project:
1.	Start Eclipse in the the workspace you created above
2.	Right-click the project you created above and select 'Refresh' to refresh the project.
Add the source folders to your project
1.	Right-click your project and select "Properties" 
2.	Select "Java Build Path" on the left
3.	Select the "Source" tab on the right
4.	Click the "Add Folder..." button on the right
5.	Check the check boxes against the folders applicable to your project: 
o	threeWorlds 
§	src/aot
§	src/threeWorlds
§	src/borisFire
6.	Click 'OK'
7.	Click 'OK'
Setup the libraries
1.	Right-click your project and select "Properties" 
2.	Select "Java Build Path" on the left
3.	Select the "Libraries" tab on the right
4.	Click the "Add Library..." button on the right
5.	Select "IvyDE Managed Dependencies" 
6.	Click 'Next'
7.	Edit the 'Ivy File' to be 'ivy.xml' located in directory 'threeWorlds'
8.	Click 'Finish'
9.	Click 'OK'
Eclipse will now automatically build the system. This will include the download and installation of required third-party jars.
In Eclipse, locate /threeWorlds/src/threeWorlds/fr/ens/biologie/threeWorlds/ui/TwMain.java
This is the main program file. Right click on the file name in the Eclipse package explorer, and select 'run as / Java application. If the application does not run and you can't fix the problem, send the error to ian.davies@anu.edu.au.
When the 3Worlds application starts, select File/Open.
Navigate to and open .3w/project_GDDM_000000000000-0000014D45B7E789-0000
In the list of model components (Models) check:
3wCore.dsl
Biology_2015a.dsl
Dispersal_2015a.dsl
Disturbance_2015a.dsl
Duration_2015a.dsl
Exp_2015a.dsl
FileLogger.dsl
GDDM.dsl
Locations_2015a.dsl
LoggerSeries.dsl
ObserverFine.dsl
In the list of User Interfaces, check:
Tab_Desktop.dsl
Widget_Disturbance.dsl
Widget_DisturbanceMean.dsl
Widget_HSN.dsl
Widget_MTDNA.dsl
Widget_Plotter.dsl
Widget_Population.dsl
Widget_SimController.dsl
Widget_StepTimer.dsl
At the top of this dialog, enter a short Configuration name (e.g. GDDM)
Click ok
The model should now be ready to run using the buttons on the “Simulaton” widget.
Before quitting, check the menu item: Widgets/Simulation/Initialise at startup. This save having to click the “initialise” button before running simulations in future.
Rearrange the widgets and resize the main window as you please. These settings are saved automatically in “./aot/Preferences.dsl”
When you restart twMain there is no need to go through all these steps again. Instead, select GDDM from the File/Recent projects/… menu list.
To alter parameter values, go to your .3w/project_GDDM_000000000000-0000014D45B7E789-0000/local/models directory and edit the relevant dsl file in a text editor.
