# Supplementary material for: Implications of recurrent disturbance for genetic diversity
Source: Ecol Evol. 2016 Jan 25;6(4):1181–96. doi: 10.1002/ece3.1948 (PMC4725449; doi:10.1002/ece3.1948)

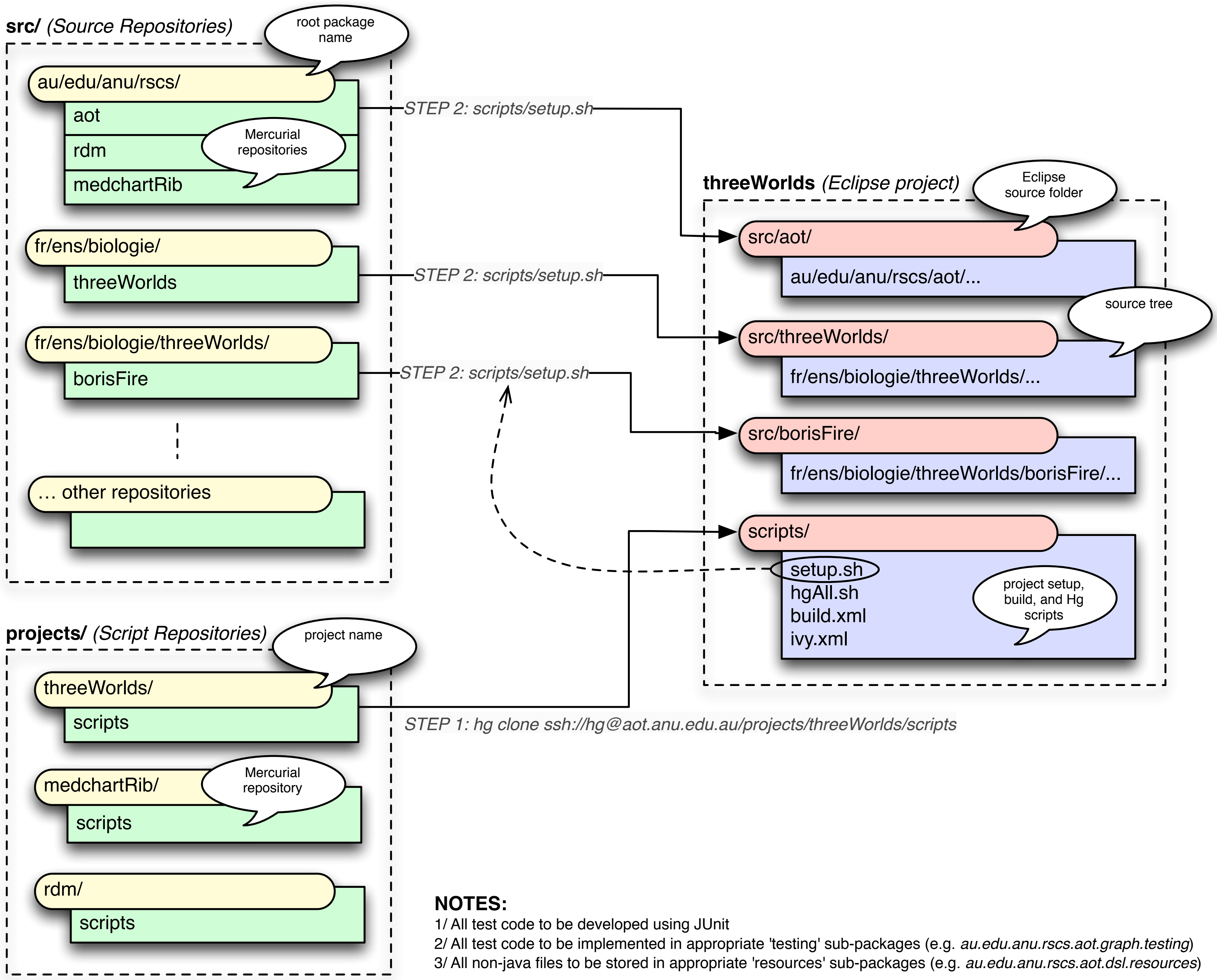

NOTES:

1/ All test code to be developed using JUnit

2/ All test code to be implemented in appropriate 'testing' sub-packages (e.g. *au.edu.anu.rscs.aot.graph.testing*)

3/ All non-java files to be stored in appropriate 'resources' sub-packages (e.g. *au.edu.anu.rscs.aot.dsl.resources*)

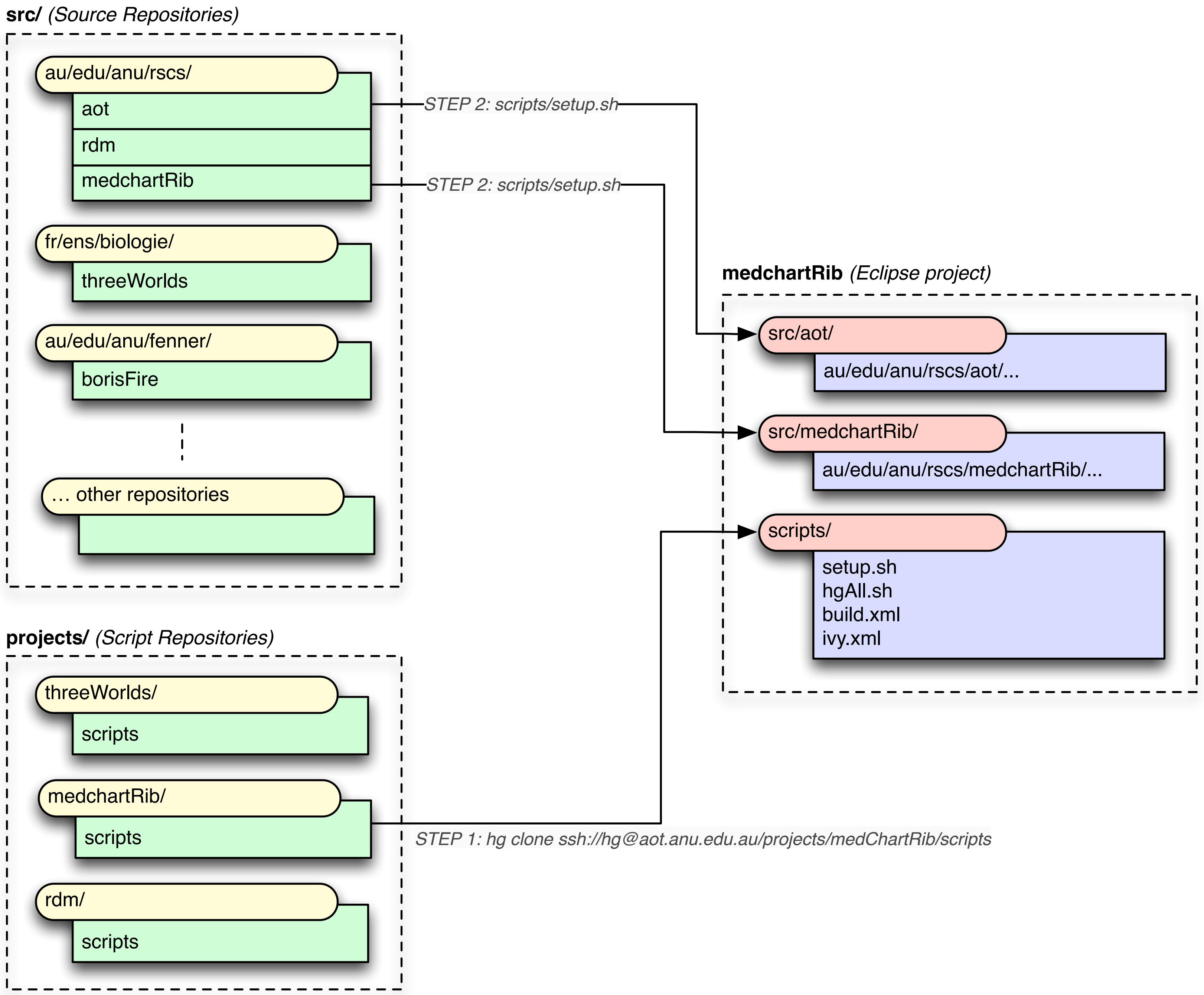

Supplement: Supplementary file 3 — Appendix S3. Java source code, parameter and input files together with installation instructions for the model used in article. [file ECE3-6-1181-s003.zip › src.zip!/src/aot/au/edu/anu/rscs/aot/doc/HgEclipseSetup.pdf]
