# Supplementary material for: Implications of recurrent disturbance for genetic diversity
Source: Ecol Evol. 2016 Jan 25;6(4):1181–96. doi: 10.1002/ece3.1948 (PMC4725449; doi:10.1002/ece3.1948)

The '**Basic**' interface attempts to hide the details of server, repository and deployment management from the user. The three indicator lights are used to summarise the status of underlying activity.
If all indicators are green, everything is OK. If not, the 'Advanced' interface will need to be used.

The '**Advanced**' interface provides complete control over the deployment and operation of an experiment.

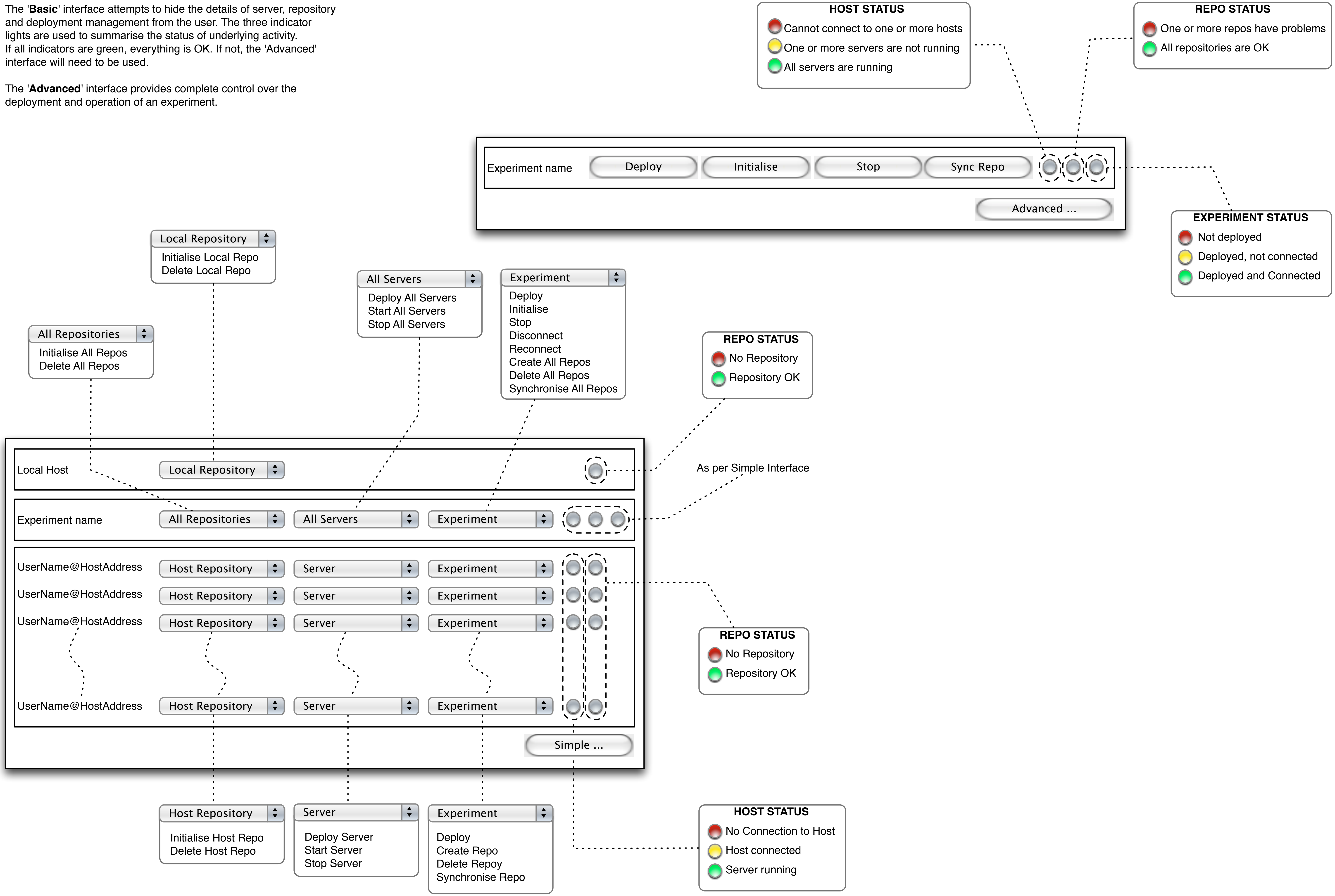

Supplement: Supplementary file 3 — Appendix S3. Java source code, parameter and input files together with installation instructions for the model used in article. [file ECE3-6-1181-s003.zip › src.zip!/src/threeWorlds/fr/ens/biologie/threeWorlds/core/computingGrid/deploymentWidget.pdf]
