# Supplementary material for: Implications of recurrent disturbance for genetic diversity
Source: Ecol Evol. 2016 Jan 25;6(4):1181–96. doi: 10.1002/ece3.1948 (PMC4725449; doi:10.1002/ece3.1948)
Supplement: Supplementary file 3 — Appendix S3. Java source code, parameter and input files together with installation instructions for the model used in article. [file ECE3-6-1181-s003.zip › src.zip!/src/threeWorlds/fr/ens/biologie/threeWorlds/thirdParty/RSyntaxTextArea-master.zip!/RSyntaxTextArea-master/src/main/java/org/fife/util/package.html]

General utility classes.
